# The Comprehension of Counterfactual Conditionals: Evidence From Eye-Tracking in the Visual World Paradigm

**DOI:** 10.3389/fpsyg.2019.01172

**Published:** 2019-06-14

**Authors:** Isabel Orenes, Juan A. García-Madruga, Isabel Gómez-Veiga, Orlando Espino, Ruth M. J. Byrne

**Affiliations:** ^1^Department of Basic Psychology I, Universidad Nacional de Educación a Distancia (UNED), Madrid, Spain; ^2^Department of Developmental and Educational Psychology (UNED), Madrid, Spain; ^3^Department of Psychology, Universidad de La Laguna, Santa Cruz de Tenerife, Spain; ^4^School of Psychology and Institute of Neuroscience, Trinity College Dublin, University of Dublin, Dublin, Ireland

**Keywords:** counterfactuals, conditionals, comprehension, visual-world-paradigm, reasoning

## Abstract

Three experiments tracked participants’ eye-movements to examine the time course of comprehension of the dual meaning of counterfactuals, such as “if there had been oranges then there would have been pears.” Participants listened to conditionals while looking at images in the visual world paradigm, including an image of oranges and pears that corresponds to the counterfactual’s conjecture, and one of no oranges and no pears that corresponds to its presumed facts, to establish at what point in time they consider each one. The results revealed striking individual differences: some participants looked at the negative image and the affirmative one, and some only at the affirmative image. The first experiment showed that participants who looked at the negative image increased their fixation on it within half a second. The second experiment showed they do so even without explicit instructions, and the third showed they do so even for printed words.

## Introduction

People often create counterfactual alternatives to reality in their everyday thoughts when they think “if only…” or “what if…” and imagine how a situation could have turned out differently (e.g., Kahneman and Tversky, 1982; Byrne, 2005). When people understand a counterfactual, such as “if there had been oranges then there would have been pears,” they appear to envisage two possibilities, the imagined alternative to reality that corresponds to the counterfactual’s conjecture, “there were oranges and pears” and the known or presumed facts that correspond to actual reality, “there were no oranges and no pears” (for a review see Byrne, 2016). In contrast, for a conditional in the indicative mood, such as “if there were oranges, then there were pears,” they tend to envisage just a single possibility at the outset, “there were oranges and pears” (e.g., Johnson-Laird and Byrne, 2002; Khemlani et al., 2018). Our aim is to examine the mental representations and cognitive processes that underpin the comprehension of counterfactuals.

Our starting point is the extensive evidence for the dual meaning of counterfactuals. To fully understand the meaning of a counterfactual, people must not only simulate the imagined alternative to reality that is conjectured in a counterfactual, they must also recover the presumed reality. But little is known about the cognitive processes that they rely on to do so (e.g., Johnson-Laird and Byrne, 1991; Byrne, 2005; Espino and Byrne, 2018). The accessibility of an imagined “possible world” from a representation of the real world poses difficulties (e.g., Stalnaker, 1968; Lewis, 1973), and what constitutes a “minimal change” is a slippery notion (e.g., Williamson, 2007; Kratzer, 2012). Nonetheless, people appear to readily recover the known or presumed facts when they understand a counterfactual. For example, participants tend to misremember a counterfactual, “if there had been oranges, then there would have been pears” and believe they were told instead, “there were no oranges and no pears” (Fillenbaum, 1974). They believe that someone uttering the counterfactual meant to imply this situation, and they judge that the items that best fit the description include this situation (e.g., Byrne and Tasso, 1999; Thompson and Byrne, 2002). Hence, the evidence indicates that they envisage the known or presumed facts, relying on their knowledge or on the cues of the subjunctive mood or content to do so.

People make more inferences that require access to “there were no oranges and no pears” from the counterfactual compared to the factual indicative conditional, such as *modus tollens* (from “there were no pears” to “therefore there were no oranges”). But they also make the same frequency of inferences that require access to “there were oranges and pears” from both conditionals, such as *modus ponens* – from “there were oranges” to “therefore there were pears” (e.g., Byrne and Tasso, 1999; Thompson and Byrne, 2002; see also Moreno-Ríos et al., 2008; Egan et al., 2009). They do so for various different sorts of content (e.g., Frosch and Byrne, 2012; see also Quelhas and Byrne, 2003; Egan and Byrne, 2012). Moreover, participants are primed to read, “there were no oranges and no pears” when they have first read the counterfactual, and they do so more quickly than when they have first read the factual conditional. But they also read, “there were oranges and pears” equally quickly from both sorts of conditional (e.g., Santamaría et al., 2005, see also Gómez-Veiga et al., 2010). Hence, the evidence indicates that people envisage both the imagined alternative to reality conjectured by the counterfactual, “there were oranges and there were pears,” and the actual reality, known or presumed by the counterfactual, “there were no oranges and there were no pears.” They keep track of their epistemic status as corresponding to real or imagined situations (Johnson-Laird and Byrne, 1991). The essence of the dual meaning of a counterfactual lies in this comparison of reality to an imagined alternative (e.g., Beck et al., 2006; Espino and Byrne, 2018). The question we wish to address is, at what point in their comprehension of a counterfactual do people detect the two messages of a counterfactual, that is, at what point do they envisage the conjecture, and at what point do they recover the presumed facts? The three experiments we report aim to advance knowledge of the comprehension of counterfactuals by establishing the point at which participants envisage each of the possibilities, during the temporal course of processing a counterfactual.

The question of when people envisage the situation corresponding to a counterfactual’s conjecture and when they envisage the presumed facts is important, first because some theories dispute that people consider both possibilities, and second, because the time at which people consider each possibility can provide a clue about the cognitive processes that they rely on to do so. Some online comprehension studies have been interpreted to support the idea that people represent both the conjecture and the presumed facts, and others have been interpreted to suggest that they represent only the conjecture. On the one hand, in eye-tracking studies it has been found that participants looked at a target word more quickly when it was presented in a context that was consistent with the real world rather than a counterfactual world. The result indicates an early and fleeting reading-time penalty that appears to reflect the construction of two representations (e.g., Ferguson and Sanford, 2008), although it may be sensitive to methodological factors (e.g., Ferguson et al., 2008, 2010; Ferguson, 2012; see also Stewart et al., 2009). Similarly, counterfactuals elicit greater brain activation, compared to factual conditionals, in areas related to conflict detection (e.g., Kulakova et al., 2013; see also Urrutia et al., 2012b). The results suggest that people represent both possibilities. But on the other hand, false counterfactuals elicit more brain activity than true ones, which may indicate the activation of only the conjecture (e.g., Nieuwland and Martin, 2012; see also Nieuwland, 2013; Kulakova and Nieuwland, 2016a,b). Accordingly, some theorists have proposed that people understand a counterfactual by considering their belief only in the imagined alternative to reality and they do so by simulating only the conjecture (e.g., Evans and Over, 2004; Evans, 2007). Hence, one view is that only the conjecture about an imagined alternative to reality is represented; another view is that both the conjecture and the presumed facts of reality are represented.

Even among theorists who propose that people consider both possibilities, there are disagreements. One theory is that the counterfactual conjecture, “there were oranges and pears” is more highly activated than the presumed facts, “there were no oranges and no pears” (e.g., Ferguson, 2012; Ferguson and Cane, 2015). An alternative theory is that when people understand a counterfactual, they first represent the presumed facts, “there were no oranges and no pears,” and the conjecture “there were oranges and pears,” although activated, does not contribute to discourse updating, is not semantically integrated, and does not remain in focus following a delay (e.g., De Vega et al., 2007; De Vega and Urrutia, 2012; Urrutia et al., 2012a). Hence, one view is that the conjecture is the more highly activated of the two possibilities, whereas another view is that the presumed facts are more highly activated. Our aim is to contribute to the resolution of these conflicting ideas.

We address a novel and nuanced question in our three experiments: if people envisage both the counterfactual’s conjecture about an imagined alternative to reality and its presumed facts, when do they do so? Our question is, at what point in the temporal process of comprehension do people consider each possibility? An answer to this question has the potential not only to distinguish between alternative theories of the comprehension of counterfactuals but also to shed light on the, at times, conflicting results of previous experiments, which have not been uniform in their choice of times at which to measure comprehension. Most importantly an answer to this question can contribute to understanding the nature of the cognitive processes by which people recover the presumed reality when they entertain the imagined alternative to reality conjectured by the counterfactual.

The three experiments we report rely on eye-tracking in the visual world paradigm to attempt to establish the time course during which people construct each possibility to understand a counterfactual. The visual world paradigm allows us to study the unfolding process of comprehending a counterfactual. In a typical visual world task, verbal and visual inputs are presented simultaneously while the participants’ eye movements are recorded to provide an index of real-time processing, sensitive to subtle aspects of language, attention, and memory (e.g., Allopenna et al., 1998; Rayner, 1998; Duñabeitia et al., 2009; Orenes et al., 2014, 2015). The logic of the method is that when something is heard, it is processed and attended to automatically; at the same time, if the corresponding object is visible, the eyes begin to move toward it (e.g., Cooper, 1974; Tanenhaus et al., 1995; for a review see Huettig et al., 2011a). It follows that when people listen to counterfactuals in a visual world paradigm, they will look more frequently at the most active information in working memory.

**Figure 1:**
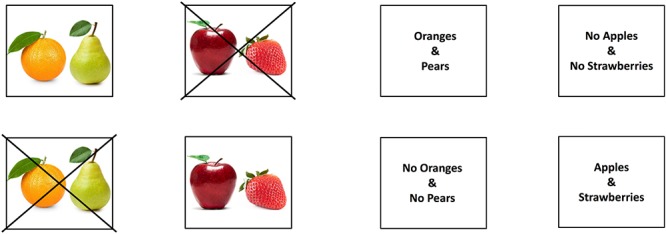
Examples of the pictorial-based visual display used in Experiments 1 and 2 (on the left), and the word-based visual display used in Experiment 3 (on the right), for the counterfactual conditional “if there had been oranges then there would have been pears.”

In the three experiments we report, participants heard short stories that contained an indicative factual conditional, e.g., “if there are oranges, then there are pears,” or a counterfactual, e.g., “if there had been oranges, then there would have been pears” (and we used a wide range of different contents, see the Supplementary Material A). In Experiment 1 and 2, four visual images related to the conditionals were shown on the screen, i.e., an image corresponding to the counterfactual’s conjecture, e.g., of oranges and pears, and an image corresponding to the presumed facts, e.g., of no oranges and no pears, as well as “distractor” images, i.e., an image corresponding to other sorts of fruit, such as apples and strawberries, and an image corresponding to no apples and no strawberries, as Figure 1 illustrates. The first two experiments differed in the instructions participants were given. In Experiment 1 participants were explicitly instructed to look at the objects on the screen that matched the meaning of the stories that they heard, in line with typical tasks in eye-tracking experiments, which encourage controlled, top-down processing. In Experiment 2 participants did not receive explicit instructions to look at the objects that matched the meaning of the stories, so that we could examine the processes underlying spontaneous counterfactual processing. In both experiments, we hypothesized that participants would look at the affirmative image, e.g., of oranges and pears, for indicative conditionals, whereas they would look at both the affirmative image and the negative image, e.g., of no oranges and no pears, for counterfactual conditionals. We anticipated that we would observe the same results with and without explicit instructions, notwithstanding an anticipated acceleration of processing of the counterfactual given explicit instructions. In Experiment 3, the same technique was used except that printed words were used instead of visual images, e.g., “oranges and pears.” We again hypothesized that participants would look at the affirmative words for indicative conditionals, whereas they would look at both the affirmative and negative words for counterfactual conditionals. We anticipated that we would observe the same results for printed words, notwithstanding once again an anticipated acceleration of processing of the counterfactual given printed words, since images can impede the comprehension of negation (e.g., Orenes and Santamaría, 2014). Our key predictions concern the temporal course of looking at the affirmative and negative images as a participant hears a counterfactual. If the recovery of the presumed reality is essential to understanding the meaning of a counterfactual, then we expect to observe a rapid increase in looking at the negative image as soon as participants detect – for example through the cues of the subjunctive mood – that the conditional conveys an imagined alternative to reality. In addition, if the essence of the dual meaning of a counterfactual is the comparison of reality to an imagined alternative, then we expect to observe that participants will maintain their gaze on both the negative and the affirmative image throughout the period of time measured.

We analyzed participants’ eye gaze at every 50 *ms* interval for a period of 4000 *ms*. One possibility is that a participant will look at only one image for a counterfactual during this time period, e.g., the affirmative image, and they will not look at the other three images. If so, the probabilities of fixations on the affirmative image will approximate 1, at every 50 *ms* “snapshot.” We anticipate this outcome to be the case for indicative conditionals. An important possibility is that participants may look at two images, the affirmative one and the negative one. Even if they rapidly and constantly switch their eye gaze from the affirmative image to the negative image and back, our snapshot of fixations every 50 *ms* will capture their gaze on one image or the other at that precise time. Hence if participants look entirely equally at both images on every trial for each counterfactual, moving their gaze from one image to the other, the probabilities of fixations on the affirmative image and on the negative image will each approximate 0.5. We anticipate this outcome to be the case for counterfactual conditionals. Of course, the same is true if a participant looks only at the affirmative image for a counterfactual on one trial, and only at the negative image for a different counterfactual on another trial. Hence, we examine not only group data but also individual data in our experiments. If participants look entirely equally at all 4 images, including the distractors, the probabilities of fixations on each one of them will each approximate 0.25, although this outcome is unlikely given that listeners tend to fixate objects that are mentioned or expected.

## Experiment 1

The aim of the experiment was to study the temporal course of the comprehension of counterfactuals. The question we wished to ask was, when people understand a counterfactual conditional, such as, “if there had been oranges then there would have been pears,” at what point in the temporal course of processing do they focus on an image corresponding to the conjecture, e.g., “there were oranges and pears” and at what point do they focus on an image corresponding to the presumed facts, e.g., “there were no oranges and no pears.” We expect that participants will begin by looking at the affirmative image for both counterfactual and indicative conditionals, since the items in the affirmative image match what is mentioned in the conditionals, but we predict that participants will exhibit a rapid increase in looking at the negative image as soon as they detect that the conditional conveys an imagined alternative to reality. We also predict that participants will continue to look at both the negative and the affirmative image throughout the measured period of time.

### Methods

#### Participants

The participants were 24 volunteers who were students at the University of La Laguna, Tenerife, Spain, and they participated in the experiment in exchange for course credits. There were 21 women and 3 men, and their average age was 20 years, with a range from 18 to 26 years. The participants were native Spanish speakers and they all reported normal vision or wore soft contact lenses or glasses.

#### Materials and Design

The design was a within-participants one and participants received vignettes in each of two conditions: indicative or counterfactual conditionals. They heard 36 vignettes about simple events (adapted from Santamaría et al., 2005), 18 trials in each of the two conditions, and the order of the trials was randomized.

The vignettes were presented to the participants in their native Spanish and started with an opening scene, e.g., “Maria went to the fruit shop to buy fruit to make a cake for Valentine’s Day. While she was waiting in the queue, she heard some clients who said” (“María fue a la frutería para comprar fruta que necesitaba para hacer un pastel por San Valentín. Mientras estaba esperando en la cola escuchó a unos clientes que decían”). The next sentence contained a conditional, either an indicative conditional, e.g., “if there are oranges, then there are pears” (“si hay naranjas, entonces hay peras”) or a counterfactual conditional, e.g., “if there had been oranges, then there would have been pears” (“si hubiera habido naranjas, entonces habría habido peras”). The following sentence contained a conjunction, either an affirmative conjunction, e.g., “María realized that there were oranges and there were pears” (“María se dio cuenta que había naranjas y había peras”) or a negative conjunction, e.g., “María realized that there were no oranges and there were no pears” (“María se dio cuenta que no había naranjas y no había peras”). The vignette ended with a closing-scene, e.g., “Finally, María also bought chocolate” (“Finalmente, María también compró chocolate”). The full set of 36 contents and their associated images is in the Supplementary Material A.

Each sentence was prerecorded and presented via speakers while four images were shown on a computer screen: two target images, e.g., an image of an orange and a pear, and an image of an orange and a pear with a cross through it, as well two distractor images, e.g., an image of other fruit such as an apple and a strawberry, and an image of an apple and a strawberry with a cross through it (see the four images on the left side of Figure 1). The position of each image (top left, top right, bottom left, bottom right quadrant) was counterbalanced across conditions. We constructed 8 versions of each vignette (e.g., oranges and pears) that varied in the conditional (indicative or counterfactual), the conjunction (affirmative or negative), and the reference to the objects in the set (e.g., oranges and pears, or apples and strawberries), as illustrated in Table 1. Each participant received only one of the 8 possible versions of each content in the set of 36 trials and the contents were assigned to the trials in a counterbalanced manner.

**Table 1:** Examples of the 8 versions of the verbal description of each content, illustrated for the oranges and pears/apples and strawberries content, for the visual display in Figure 1.

(1) Indicative affirmative	Oranges	If there are oranges, then there are pears. María realized that there were oranges and there were pears.
	Apples	If there are apples, then there are strawberries. María realized that there were apples and there were strawberries.
(2) Indicative negative	Oranges	If there are oranges, then there are pears. María realized that there were no oranges and there were no pears.
	Apples	If there are apples, then there are strawberries. María realized that there were no apples and there were no strawberries.
(3) Counterfactual affirmative	Oranges	If there had been oranges, then there would have been pears. María realized that there were oranges and there were pears.
	Apples	If there had been apples, then there would have been strawberries. María realized that there were apples and there were strawberries.
(4) Counterfactual negative	Oranges	If there had been oranges, then there would have been pears. María realized that there were no oranges and there were no pears.
	Apples	If there had been apples, then there would have been strawberries. María realized that there were no apples and there were no strawberries.

#### Apparatus and Procedure

Participants listened to the 36 stories over speakers while looking at a computer screen and at the end of each story they answered a simple question about it. Participants were instructed that they should listen to the sentences carefully and that they should not take their eyes off the screen throughout the experiment. They were explicitly instructed to look at the object or objects on the screen that matched the meaning of the stories that they heard. These explicit instructions were based on the usual information provided to participants in eye-tracking experiments, which typically specify how to interact with the display, e.g., by touching, clicking, or moving objects. Their eye movements were recorded at a rate of 500 Hz using an SR Research EyeLink II head-mounted eye-tracker connected to a 21 color CRT for visual stimulus presentation. Procedures were implemented in SR Research Experiment Builder. Calibration and validation procedures were carried out at the beginning of the experiment and were repeated several times per session. Trials started with the presentation of a central fixation dot for drift correction while participants listened to the opening scene sentence. Next, a display with four images appeared for 2 s. Then the story began, and the images remained on screen for the entire time while the remainder of the story was heard over speakers. The trial concluded with the appearance of a simple question on the screen, e.g., “Did María go to the fruit shop?” (“¿Fue María a la frutería?”)^1^, which participants answered by pressing either a “yes” or a “no” button on a game-pad, by pressing with their right index finger for yes and their left index finger for no. There was a practice block of four trials before the experiment proper started. The experiment lasted approximately 30–40 min and each participant was tested individually.

### Results and Discussion

The data for the three experiments is available at https://reasoningandimagination.com/data-archive/ and on OSF at https://osf.io/n6hk3/. Prior to any data analysis one participant was eliminated from the analysis because her eye-movements explored the screen continuously without any systematic fixations on any point.

#### Eye-Tracking Data Coding

The eye-movement data generated by the EyeLink system were analyzed as follows. First, bitmap templates were created for identifying regions of interest in each display (the four pictures of the screen, e.g., oranges and pears, no oranges and no pears, apples and strawberries, and no apples and no strawberries). The object regions were defined in terms of rectangles containing the relevant objects, fixations landing within the perimeters of these rectangles were coded as fixations on the relevant objects. The output of the eye-tracker included the x- and y-coordinates of participant fixations, which were converted into region of interest codes using the templates.

The analysis of fixations was time-locked to the onset of the first object in the conditional, e.g., the onset of “oranges” in “if there *are/had been* oranges” and continued to 4000 *ms* after that word, which included listening to the rest of the conditional, “then there *are/would have been* pears” followed by a silent period. The periods were divided into 50 *ms* time slots and for each time slot, the number of fixations on each rectangle quadrant of the image was counted and converted into fixation probabilities^2^.

To avoid problems inherent in proportional data, participant averages were arcsin-transformed prior to *t*-test comparisons. Given that 180–200 *ms* are usually assumed to account for saccade programming (Martin et al., 1993), the mean of the first time-region (0–100 *ms*) was considered to be the baseline and was used to conduct statistical comparisons against means on each time points at 50 *ms* intervals until 4000 *ms* later (for a similar method, see Huettig and Altmann, 2011). This correction to baseline allowed us to control for any bias in the pattern of fixations on images caused by the type of context. A false discovery rate (FDR) thresholding procedure, referred to as *pFDR-corr*, was used as an alpha correction to control for Type 1 errors due to multiple comparisons (81 for each condition; see Genovese et al., 2002).

#### *T*-Tests Against Baseline

**Figure 2:**
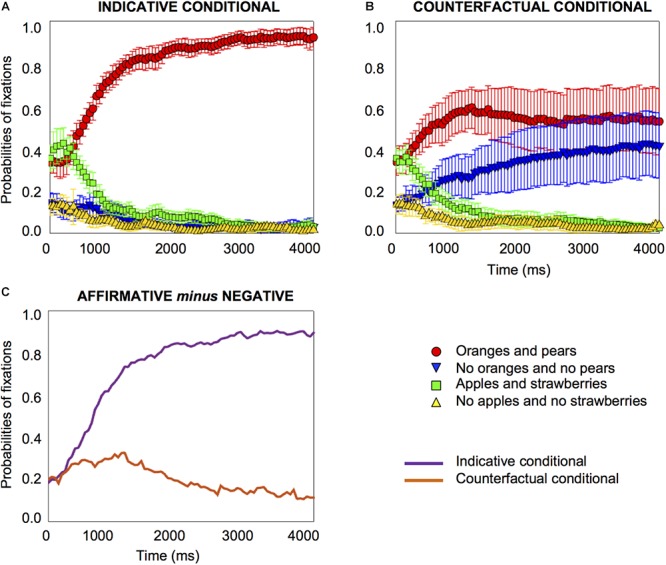
Probabilities of fixations for indicative conditionals, e.g., “if there are oranges, then there are pears” **(A)** and counterfactual conditionals, e.g., “if there had been oranges, then there would have been pears” **(B)** in Experiment 1 on the affirmative and negative images, and the two distractor images, time-locked to the onset of the first object word, e.g., oranges. The differences in the probabilities of fixations (on the affirmative image minus the negative image) for indicative and counterfactual conditionals emerge at 850 *ms*, as **(C)** shows. Error bars are 95% confidence intervals within participants (see Morey, 2008; O’Brien and Cousineau, 2014).

The results reveal that participants looked at very different parts of the visual images on screen when they heard an indicative conditional compared to when they heard a counterfactual conditional, as Figure 2 shows. For indicative conditionals, at the onset of the target word (e.g., oranges), participants were focused on the affirmative image (oranges and pears) and the affirmative distractor (apples and strawberries) about equally frequently, with probabilities of fixation of about 0.4, as Figure 2A shows. This starting point may merely reflect a tendency to look at what is present rather than what is not present. What is revealing is that very early on in the process, 450 *ms* after the target word onset, the probabilities of fixation on the affirmative image started to increase (*pFDR-corr* = 0.002); fixations decreased on all other images, including the negative image (from 350 *ms*, *pFDR corr* = 0.034) (see the Supplementary Material B for details of the comparisons for the distractor images). The results show that for an ordinary indicative conditional, participants increase their fixation on the affirmative image very early indeed in the temporal course of processing, and fixations on the other three images decrease rapidly, as Figure 2A shows. Their fixation on the affirmative image continued throughout the period of measurement to 4000 *ms*.

A very different pattern emerges for counterfactual conditionals, e.g., “if there had been oranges, then there would have been pears.” At the onset of the target word, e.g., “oranges,” participants were focused on the affirmative image (oranges and pears) and the affirmative distractor (apples and strawberries) equally frequently with probabilities of fixations of about 0.4, as Figure 2B shows. Most revealingly, from very early on, 300 *ms* after the target word onset, the probabilities of fixation on the affirmative image started to increase (*pFDR-corr* = 0.039), and fixation on the negative image also *increased* (from 650 *ms*, *pFDR-corr* = 0.031). The results show that early in the temporal course of processing, within about half a second, participants increase their fixation not only on the affirmative image but also on the negative image; fixations on the two distractor images decrease rapidly, as Figure 2B shows (see the Supplementary Material B for details about the distractor images). Equally importantly, their fixation on both images continues throughout the period of measurement to 4000 *ms*.

#### Growth-Curve Analysis

We carried out a growth-curve analysis (Mirman, 2014; see the Supplementary Material B for details) which showed that people looked at the affirmative image more for the indicative conditional than the counterfactual, and they looked at the negative image more for the counterfactual than the indicative conditional. The increase of fixations on the affirmative image occurred more quickly for the indicative than the counterfactual conditional, and the opposite was the case for the negative image.

#### Analysis by Items

As a check that each of the 36 contents was interpreted in essentially the same way, we also carried out a similar analysis to compare indicative and counterfactual conditionals with *t*-tests against the baseline, but this time by items rather than by participants. It showed the same results. For indicative conditionals, at the onset of the target word (e.g., oranges), participants’ focus was on the affirmative image and the affirmative distractor equally frequently. From 450 *ms* after the target word onset, the probabilities of fixation on the affirmative image started to increase (*pFDR-corr* = 0.003); fixations on the other images decreased, including for the negative image (from 1050 *ms*, *pFDR-corr* = 0.034) (see the Supplementary Material B for details about the distractor images).

**Figure 3:**
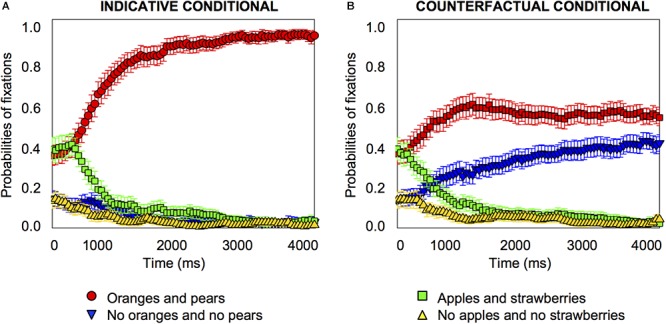
Item-analysis probabilities of fixations for indicative conditionals, e.g., “if there are oranges, then there are pears” **(A)** and counterfactual conditionals, e.g., “if there had been oranges, then there would have been pears” **(B)** in Experiment 1. Error bars are 95% confidence intervals within items.

For counterfactual conditionals, at the onset of the target word, the focus was on the affirmative image and the affirmative distractor equally frequently. From 300 *ms* after the target word onset, the probabilities of fixation on the affirmative image started to increase (*pFDR-corr* = 0.013) and so did fixation on the negative image (from 500 *ms*, *pFDR-corr* = 0.017), as Figure 3 shows. It is noteworthy that the 95% confidence interval error is very much reduced for the counterfactuals in the by-item analysis compared to the by-participants one, as a glance at Figure 2B and 3B shows, which suggests that the variance originates in differences between participants rather than differences between items. Accordingly, we also carried out an analysis of individual differences.

#### Individual Differences Analysis

**Figure 4:**
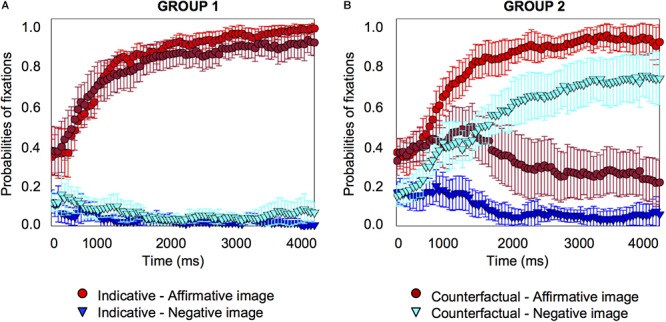
Individual differences probabilities of fixations for indicative and counterfactual sentences for one subset group of 11 participants who looked at the affirmative image for counterfactuals **(A)**, and a second subset group of 12 participants who looked at the negative image only or at the affirmative and negative image for counterfactuals **(B)** in Experiment 1. Error bars are 95% confidence intervals within participants.

We plotted individual graphs for each of the 23 participants, which are provided in the Supplementary Material C. As these graphs show, about half of the participants (*n* = 11) tended to look at the affirmative image only when they heard the counterfactual, just as they did for the indicative conditional; the other half of the participants (*n* = 12) tended to look at the negative image only (seven participants), or at both the affirmative and the negative image (five participants) for the counterfactual. We combined participants who looked at the negative image only and those who looked at the affirmative and negative image into a single sub-set group because consideration of the negative image (corresponding to the presumed facts of a counterfactual) indicates that individuals have reached a counterfactual interpretation of the conditional (and there are in any case too few participants to create three separate groups for reliable statistical analysis). These two sub-set groups of participants, affirmative only versus negative or negative-plus-affirmative, exhibited very different fixation patterns on the affirmative and negative image, as Figure 4 shows.

Both groups showed similar patterns for the indicative conditional, the probabilities of fixation on the affirmative image started to increase early on (group 1 from 450 *ms*, *pFDR-corr* = 0.025; group 2 from 450 *ms*, *pFDR-corr* = 0.020), and to decrease on the other three images, including the negative image (group 1 from 800 *ms*, *pFDR-corr* = 0.037; group 2 from 1200 *ms*, *pFDR-corr* = 0.034) (see the Supplementary Material B for details about the distractor images). However, the two groups showed different patterns for the counterfactual. Group 1’s pattern was the same as for the indicative: probabilities of fixation on the affirmative image started to increase early on (from 350 *ms*, *pFDR-corr* = 0.030), and probabilities of fixations on the other three images decreased, including for the negative image (from 1450 *ms*, *pFDR-corr* = 0.023). Group 2’s pattern was different: probabilities of fixation for the affirmative image showed no significant changes to the baseline, but they *increased* for the negative image (from 400 *ms*, *pFDR-corr* = 0.039).

The analysis shows that when people understand the indicative conditional, they look at the affirmative image from very early (450 *ms*) and decrease their fixations on the negative image quite some time later (800 *ms* in Group 1; 1200 *ms* in Group 2). When they understand the counterfactual, one subset of participants do the same thing as for the indicative, they look at the affirmative image from very early (350 *ms*) and decrease their fixations on the negative image quite some time later (1450 *ms*); however, the other subset of participants look at the affirmative image early and continue to do so at the same rate as at the baseline throughout, but these participants look increasingly at the negative image and from very early indeed (400 *ms*).

The experiment provides information on the points in the temporal course of processing a counterfactual, e.g., “if there had been oranges, then there would have been pears,” when people focus on an image corresponding to the conjecture, e.g., “there were oranges and pears” and on an image corresponding to the presumed facts, e.g., “there were no oranges and no pears.” The results show that when people understand an indicative conditional, e.g., “if there are oranges, then there are pears,” shortly after they hear the word “oranges,” their focus increases on the affirmative image, and their focus on the other three images decreases fairly rapidly. The overall group results for indicative conditionals are reflected also in the results for each individual. The results show a different pattern for counterfactuals. The results averaged over the whole group of participants show that when they understand the counterfactual, e.g., “if there had been oranges, then there would have been pears,” very early in the temporal course of processing, they increase their focus not only on the affirmative image but also on the negative image, and this focus on both images continues throughout the period of measurement. However, there are pronounced individual differences. About half of the participants appear to understand the counterfactual just as they do the indicative conditional, and they focus on the affirmative image only. The other half of the participants understand the counterfactual differently from the indicative conditional – they continue to look at the affirmative image as much as they do at the outset, but they increase their focus on the negative image.

One possible explanation for the individual differences is that the instruction to look at what the stories mean may be interpreted by some participants to look at what is explicitly mentioned in the counterfactual, e.g., oranges and pears, whereas it may be interpreted by others to look at what is presumed by the counterfactual, e.g., no oranges and no pears. To rule out this possibility, we carried out a second experiment with the aim of testing whether the results are replicated when participants are not given this explicit instruction.

## Experiment 2

The aim of the experiment was to test whether the results of the previous experiment are replicated in an implicit task, that is, when participants are *not* given an explicit instruction to look at the object or objects on the screen that matched the meaning of the stories that they heard. In this way we aimed to examine further the spontaneous or automatic processes underlying the comprehension of counterfactuals.

### Methods

#### Participants

The participants were a new set of 24 native Spanish speakers from the University of La Laguna, Tenerife, Spain, who participated in the experiment in exchange for course credits. There were 15 women and 9 men, and their average age was 19 years, with a range from 18 to 23 years. All of them reported normal vision or wore soft contact lenses or glasses.

#### Materials, Design and Procedure

The materials, design and procedure were the same as Experiment 1. The only difference was that participants were *not* instructed to look at the object or objects on the screen that matched the meaning of the stories they heard, as the participants in the previous experiment had been instructed. Participants were instructed to listen to the sentences and answer the simple question at the end. They were also told not to take their eyes off the screen throughout the experiment.

### Results and Discussion

One participant was eliminated from the analysis because she looked at just one point on the screen throughout the experiment and no moves were registered for her, and five participants were eliminated because they explored the screen continuously without fixations on any point. The procedure for analyzing the eye movement data was the same as that used in the previous experiment.

#### *T*-Tests Against Baseline

**Figure 5:**
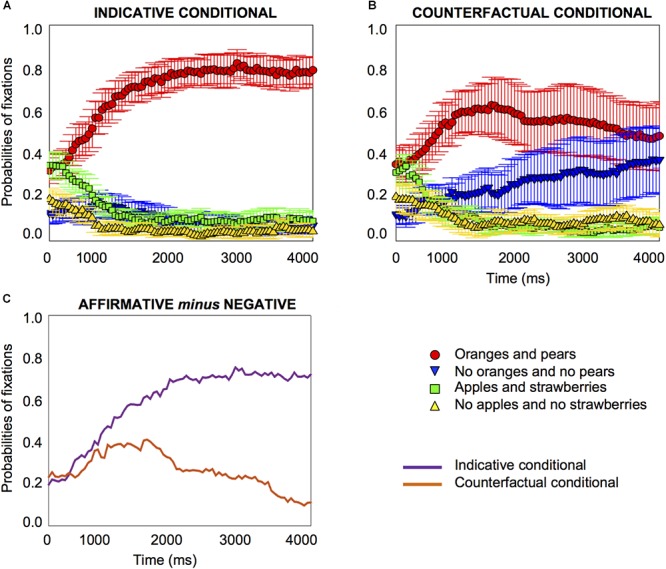
Probabilities of fixations for indicative conditionals, e.g., “if there are oranges, then there are pears” **(A)** and counterfactual conditionals, e.g., “if there had been oranges, then there would have been pears” **(B)** in Experiment 2. The differences in the probabilities of fixations (on the affirmative image minus the negative image) for indicative and counterfactual conditionals emerge at 1700 ms, as **(C)** shows. Error bars are 95% confidence intervals within participants.

The results replicated the previous experiment, as Figure 5 shows. For the indicative conditional, at the onset of the target word, participants were focused on the affirmative image and the affirmative distractor equally frequently, with a probability of 0.3 to 0.4. From 400 *ms* after the target word onset, the probabilities of fixation on the affirmative image started to increase (*pFDR-corr* = 0.023); no significant change was observed for the negative image, as Figure 5A shows (see Supplementary Material B for details about the distractor images). Hence, participants looked at the affirmative image for the indicative conditional, replicating the findings of the previous experiment.

For the counterfactual, at the onset of the target word, participants were focused on the affirmative image and the affirmative distractor equally frequently, with a probability of 0.3 to 0.4. After the target word onset, there was an increase in fixations on the affirmative image (from 550 *ms*, *pFDR-corr* = 0.033), and an *increase* on the negative image (from 450 *ms*, *pFDR-corr* = 0.034). Hence, participants looked at the affirmative and the negative image for the counterfactual, replicating the findings of the previous experiment, as Figure 5B shows.

#### Growth-Curve Analysis

The growth curve analysis showed the same results as the previous experiment (see the Supplementary Material B for details). However, as Figure 5C shows, the differences between the indicative and counterfactual conditionals emerge at 1700 *ms*, which is considerably later than in the previous experiment (850 *ms*). Participants maintained their gaze on the affirmative image for both types of conditional until the negative image started to be fixated at a later time. This result reflects the difference in instructions between the two experiments and indicates that the instruction in the previous experiment to look at the objects that correspond to what the sentence means resulted in an earlier focus on the negative image in the understanding of the counterfactuals.

The results provide information on how people understand indicative and counterfactual conditionals and also reveal important clues about the implicit processes in the comprehension of counterfactuals, without explicit instruction. Despite the absence of instruction to look at the objects corresponding to what the sentence means, there is consistency in the results of this experiment and the previous one. The results suggest that people automatically look at the images that correspond to what they understand in this situation; when they are given instructions to do so explicitly, their processing of the sentences is accelerated, but the processing nonetheless remains the same.

#### Individual Differences Analysis

**Figure 6:**
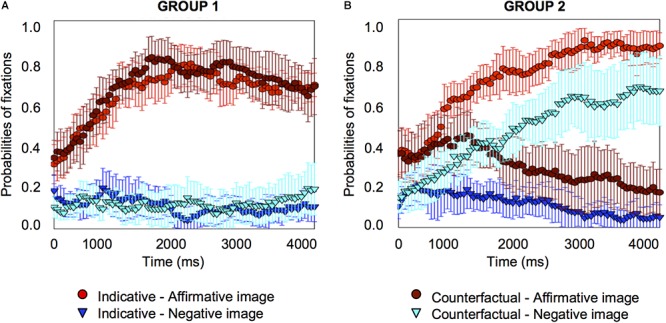
Individual differences probabilities of fixations for indicative and counterfactual sentences for a subset group of 10 participants who looked at the affirmative image for counterfactuals **(A)**, and a subset group of eight participants who looked at the negative image only or at the affirmative and negative image for counterfactuals **(B)** in Experiment 2. Error bars are 95% confidence intervals within participants.

We again plotted individual graphs for each of the 18 participants, which are provided in the Supplementary Material C. Once again about half of the participants looked at the affirmative image only, when they heard the counterfactual (10 participants), just as they did for the indicative conditional; the other half looked at the negative image (eight participants, four who looked at the negative image and four who looked at both the negative and the affirmative image), as Figure 6 shows.

For the indicative conditional, both groups showed the same pattern: the probabilities of fixation on the affirmative image increased (group 1 from 250 *ms*, *pFDR-corr* = 0.044; group 2 from 750 *ms*, *pFDR-corr* = 0.022) and fixations on the other images decreased, including for the negative image (group 1, no significant change; group 2 from 300 *ms*, *pFDR-corr* < 0.001) (see the Supplementary Material B for details about the distractor images). For the counterfactual, the groups showed different patterns. For group 1, the pattern was the same as the indicative conditional, the probabilities of fixation on the affirmative image increased (from 350 *ms, pFDR-corr* = 0.029), for the negative image there was no significant change. Group 2’s pattern was different: probabilities of fixation for the affirmative image showed no significant changes to the baseline, but they *increased* for the negative image (from 700 *ms, pFDR-corr* = 0.027). The results are consistent with the previous experiment.

The experiment replicates and extends the findings of the previous experiment. The results show that about half of the participants in both experiments tend to look at both the affirmative and the negative image when they hear a counterfactual, or at the negative image; the other half look only at the affirmative image, just as they do for the indicative conditional. In the next experiment we extend the findings to a verbally based visual world paradigm.

## Experiment 3

The aim of the experiment was to test whether the results of the previous experiments are replicated, this time for a verbally based visual world paradigm. The experiment had the same design as the previous experiments, but printed words were shown instead of pictures, as Figure 1 shows (on the right-hand side). Most of the studies that compare both formats show similar results for them (e.g., McQueen and Viebahn, 2007; Primativo et al., 2016). The printed word version may be more sensitive to phonological manipulations than the traditional picture version (e.g., Huettig and McQueen, 2011, see also Weber et al., 2007). The printed word version is useful for investigating orthographic processing during speech perception but less so for investigating processing of semantic and conceptual visual-form representations (Salverda and Tanenhaus, 2010; Huettig and McQueen, 2011). However, visual information such as pictures has been found to impede relational and conditional reasoning, as well as reasoning about negation (e.g., Knauff and Johnson-Laird, 2002; Orenes and Santamaría, 2014). Hence our aim was to examine whether the same results occur for the printed word version as for the pictures version of the visual world paradigm. We also aimed to rule out any possibility that the negative visual images used in the previous experiments, e.g., an orange and a pear with a cross through it, was confusing for participants. Participants were explicitly instructed to look at the object or objects on the screen that matched the meaning of the stories that they heard, as they were in Experiment 1.

### Methods

#### Participants

The participants were a new set of 24 native Spanish speakers from the University of La Laguna, Tenerife, Spain, who participated in the experiment in exchange for course credits. There were 22 women and 2 men, and their average age was 20 years, with a range from 18 to 41 years. All of them reported normal vision or wore soft contact lenses or glasses. None of them had taken part in the previous experiments.

#### Materials, Design and Procedure

The materials, design and procedure were the same as the previous experiments. The only difference was that we presented printed words instead of images on screen, as shown in Figure 1. Participants were explicitly instructed to look at the object or objects on the screen that matched the meaning of the stories that they heard, as in Experiment 1.

### Results and Discussion

Prior to any data analysis the data of two participants were discarded because one participant fixated on just one point on the screen throughout the experiment and no eye-movements were registered, and the other participant had too many blinks. The procedure for analyzing the eye movement data was the same as that used in the previous experiments.

#### *T*-Tests Against Baseline

**Figure 7:**
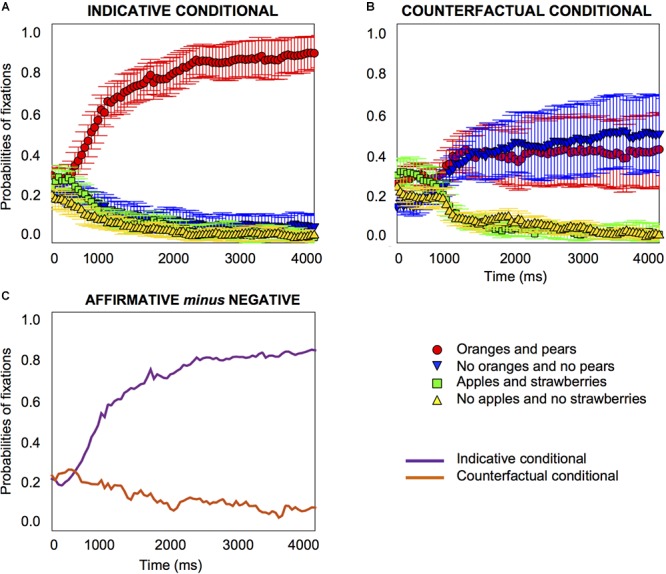
Probabilities of fixations for indicative conditionals, e.g., “if there are oranges, then there are pears” **(A)** and counterfactual conditionals, e.g., “if there had been oranges, then there would have been pears” **(B)** in Experiment 3. The differences in the probabilities of fixations (on the affirmative printed words minus the negative printed words) for indicative and counterfactual conditionals emerge at 550 *ms*, as **(C)** shows. Error bars are 95% confidence intervals within participants.

The results replicated the previous experiments, as Figure 7 shows. For the indicative conditional, at the onset of the target word, participants were focused on the affirmative printed words and the affirmative distractor equally frequently. From 450 *ms* after the target word onset, the probabilities of fixation on the affirmative printed words started to increase (*pFDR-corr* = 0.027), fixations on the other printed words decreased, including for the negative printed words (from 800 *ms*, *pFDR-corr* = 0.041) (see the Supplementary Material B for details about the distractor images). The results replicate the findings of the previous experiments that participants increase their fixation on the affirmative printed words very early on, and fixations on the other three printed words, including the negative printed words, decrease rapidly, as Figure 7A shows.

For the counterfactual, at the onset of the target word, participants were focused on the affirmative printed words and the affirmative distractor equally frequently. After the target word onset, fixations on the affirmative printed words remained at about 0.3 to 0.4 and did not change; from 450 *ms* there was an *increase* in fixation on the negative printed words (*pFDR-corr* = 0.039). The results replicate those of the previous experiments that early in the temporal course of processing a counterfactual, within about half a second, participants fixate increasingly on the negative printed words, their fixation on the affirmative printed words did not change from the baseline, and fixations on the two distractors decrease rapidly, as Figure 7B shows.

#### Growth-Curve Analysis

The growth curve analysis showed the same results as the previous experiments (see the Supplementary Material B for details).

#### Individual Differences Analysis

**Figure 8:**
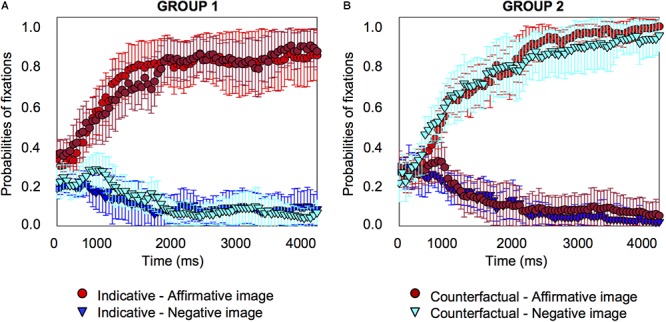
Individual differences probabilities of fixations for indicative and counterfactual sentences for the subset group of nine participants who looked at the affirmative printed words for counterfactuals **(A)**, and the second subset group of 13 participants who looked at the negative printed words only or at the affirmative and negative printed words for counterfactuals **(B)** in Experiment 3. Error bars are 95% confidence intervals within participants.

The Supplementary Material C provides the individual graphs for each of the 22 participants. Almost half of the participants looked at the affirmative printed words only, when they heard the counterfactual (nine participants), just as they did for the indicative conditional; more than half looked at the negative printed words (13 participants, 8 who looked at the negative printed words and 5 who looked at both the negative and the affirmative printed words) as Figure 8 shows.

For the indicative conditional, both groups showed the same pattern: the probabilities of fixation on the affirmative printed words increased (group 1 from 400 *ms*, *pFDR-corr* = 0.040; group 2 from 600 *ms*, *pFDR-corr* = 0.033) and fixations on the other printed words decreased, including for the negative printed words (group 1 from 1400 *ms*, *pFDR-corr* = 0.032; group 2 from 950 *ms*, *pFDR-corr* = 0.039) (see the Supplementary Material B for details about the distractor images). For the counterfactual, the groups showed different patterns. For group 1, the pattern was the same as the indicative conditional, the probabilities of fixation on the affirmative printed words increased (from 500 *ms*, *pFDR-corr* = 0.037), and fixations on the other printed words decreased, including for the negative printed words (from 1850 *ms*, *pFDR-corr* = 0.025). Group 2’s pattern was different: probabilities of fixation for the affirmative printed words showed no significant changes to the baseline, but they *increased* for the negative printed words (from 250 *ms*, *pFDR-corr* = 0.046).

The analysis shows that when people understand the indicative conditional, they look at the affirmative printed words from very early (400–600 *ms*) and decrease their fixations on the negative printed words quite some time later (1400 *ms* in Group 1; 950 *ms* in Group 2). When they understand the counterfactual, one subset of participants do the same thing as for the indicative, they look at the affirmative printed words from very early (500 *ms*) and decrease their fixations on the negative printed words quite some time later (1850 *ms*), but the other subset of participants look at the affirmative printed words early and continue to do so at the same rate as the baseline throughout, but these participants look increasingly at the negative printed words from very early (250 *ms*). The pattern of a subset of participants focusing on the counterfactual negative printed words is particularly clear-cut for the printed word version of the visual world paradigm.

The experiment replicates and extends the findings of the previous experiments when participants are provided with the printed word version of the visual world paradigm. Therefore, the results rule out the possibility that participants were confused in the previous experiments by the representation of the absence of objects, such as “no oranges and no pears,” by an image of the objects with a cross through it, or that they experienced other difficulties in identifying the objects. Once again, the overall group data show that when people understand the counterfactual, e.g., “if there had been oranges then there would have been pears,” very early in the temporal course of processing, they increase their focus on the negative printed words overall, and continue to maintain their focus on the affirmative printed words at the same rate as at the baseline, and this focus on both sorts of printed words continues throughout the period of measurement. The experiment again shows pronounced individual differences. About half of the participants appear to understand the counterfactual just as they do the indicative conditional, they focus on the affirmative printed words only. The other half of the participants understand the counterfactual differently from the indicative conditional, they focus on the affirmative and the negative printed words.

#### Individual Differences Analysis Over the Three Experiments

**Figure 9:**
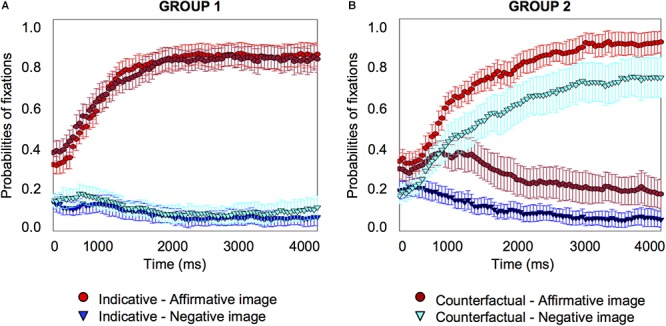
Individual differences probabilities of fixations for indicative and counterfactual sentences for the subset group of 30 participants who looked more frequently at the affirmative image or printed words for counterfactuals **(A)**, and the second subset group of 33 participants who looked more frequently at the negative one for counterfactuals **(B)** in all experiments. Error bars are 95% confidence intervals within participants.

To increase the power of the individual differences analysis, we combined the data from the 63 participants who took part in the three experiments, since they were drawn from the same population. Given that the experiments used the same materials and the results were similar, we carried out an exploratory cluster analysis k-mean to discover similarities in participants’ patterns of counterfactual processing. The analysis split participants into two subgroups depending on how they processed counterfactuals. From the combined participant set, 30 participants looked at the affirmative image (or printed words) more frequently than the negative one when they heard the counterfactual (the 11 participants described earlier from experiment 1, 10 from experiment 2, and 9 from experiment 3), and 33 participants looked at the affirmative image (or printed words) less frequently than the negative one (12 participants from experiment 1, 8 from experiment 2, and 13 from experiment 3) as Figure 9 shows.

For the indicative conditional, both groups showed the same pattern: the probabilities of fixation on the affirmative image (or printed word) increased (group 1 from 350 *ms, pFDR-corr* = 0.008; group 2 from 450 *ms*, *pFDR-corr* = 0.034) and fixations on the other images/printed words decreased, including for the negative image/printed word (group 1 from 1300 *ms*, *pFDR-corr* = 0.034; group 2, from 900 *ms, pFDR-corr* = 0.023) (see the Supplementary Material B for details about the distractor images). For the counterfactual, the groups showed different patterns. For group 1, the pattern was the same as the indicative conditional, the probabilities of fixation on the affirmative image (or printed word) increased (from 300 *ms*, *pFDR-corr* = 0.020), and fixations on the other images/printed words decreased, including for the negative image/printed word (from 1200 *ms*, *pFDR-corr* = 0.030). Group 2’s pattern was different: probabilities of fixation did not change for the affirmative image/printed word, but they *increased* for the negative image/printed word (from 250 *ms*, *pFDR-corr* = 0.044).

The analysis shows that when people understand the indicative conditional, they look at the affirmative image or printed words from very early (350–450 *ms*) and decrease their fixations on the negative image (or printed word) quite some time later (1300 *ms* in Group 1; 900 *ms* in Group 2). When they understand the counterfactual, one subset of participants do the same thing as for the indicative, they look at the affirmative image or printed words from very early (300 *ms*) and decrease their fixations on the negative image/printed word quite some time later (1200 *ms*), but the other subset of participants look at the affirmative image or printed words early and continue to do so at the same rate as the baseline throughout, and these participants increasingly look at the negative image/printed word and from very early on (250 *ms*). The results show that the previous individual differences analyses hold for the combined larger sample size.

#### Growth-Curve Analysis

We carried out a growth curve analysis of the combined data from the three experiments (see the Supplementary Material B for details). As Figure 9 shows, for group 1, there were no differences between indicative conditionals and counterfactuals; but for group 2, there were differences for both types of conditional. For the indicative conditional participants in group 2 increase their focus on the affirmative image (or printed word) during the time period measured whereas for the counterfactual conditional they show no significant increase or decrease in their focus on the affirmative image/printed word, and hence they looked at the affirmative image/printed word more for the indicative conditional than for the counterfactual. Moreover, for the counterfactual they increased fixations on the negative image (or printed word) very early, around 250 *ms*, and hence they looked at the negative image/printed word more for the counterfactual than for the indicative conditional. The result confirms the growth curve analyses for each of the three experiments (see the Supplementary Material B for details).

## General Discussion

Our objective was to explore the unfolding processing of counterfactual conditionals over time, to test the theory that to fully understand a counterfactual conditional, people must imagine the alternative to reality that it conjectures, and they must also recover the known or presumed reality. The three experiments provide information on the temporal course of processing indicative conditionals such as, ‘if there are oranges, then there are pears’ and counterfactual conditionals, such as, “if there had been oranges, then there would have been pears,” by examining the affirmative and negative images that people look at in a visual world display when they hear such conditionals.

We have discovered striking differences in what people look at when they hear counterfactuals and indicative conditionals, as revealed by the analyses of the overall group data in the three experiments. However, we have also discovered notable individual differences in how people understand counterfactuals. For the comprehension of an indicative conditional, e.g., “if there are oranges, then there are pears,” the overall group results reflect the results for the individual participants: each participant exhibited a very rapid focus on the affirmative image or printed word of oranges and pears, within half a second of hearing the target word, e.g., “oranges” (and this focus occurred at about 400 to 450 *ms* in the three experiments). Their focus on the affirmative image or printed word was accompanied by a rapid *decrease* in focus on the negative image or printed word of no oranges and no pears (occurring at about 350 to 800 *ms* in the three experiments).

For the comprehension of a counterfactual conditional, e.g., “if there had been oranges then there would have been pears,” the overall group results reflect the results of one subset of the participants, comprising about half the sample. The overall group data show a very rapid focus, within half a second, on the affirmative image or printed word corresponding to the conjecture, “there were oranges and there were pears” (occurring at about 300–550 *ms* in the three experiments). Strikingly, the focus on the affirmative image or printed word is matched by a rapid *increase* in focus on the negative image or printed word corresponding to the presumed facts, e.g., “there were no oranges and no pears” (occurring at about 450–650 *ms* in the three experiments). Thus, the results from the overall group data indicate that the comprehension of the dual meaning of counterfactuals emerges very rapidly, and participants focus on the affirmative and the negative image within about half a second of hearing the target word, e.g., “oranges” at the end of the antecedent clause. But the individual differences analyses show that these differences are due to one subset of participants. About half of the participants understood the counterfactual differently from the indicative conditional – their focus on the affirmative image or printed word showed no significant increase or decrease from their baseline tendency throughout the time period, but they *increased* their fixations on the negative image or printed word for the counterfactual conditional at a strikingly early time point, from 250 to 700 *ms*. But the other half of the participants in the three experiments appeared instead to understand the counterfactual just as they did the indicative conditional, and they focused only on the affirmative image or printed word (from 350–500 *ms* in the three experiments). They looked only at the affirmative image even for the counterfactual, and they tended to decrease their fixations on the negative image quite late in the temporal process of comprehension (from 1450 to 1850 *ms* in the three experiments).

The results for the overall group data, and for the subset of half of the participants who focused on the negative image or printed word for a counterfactual, corroborate the prediction of a rapid increase in looking at the negative image or printed word as soon as participants detected that the counterfactual communicates an imagined alternative to reality. This finding supports the theory that the recovery of the presumed reality is essential to the full understanding of the meaning of a counterfactual (e.g., Johnson-Laird and Byrne, 1991; Espino and Byrne, 2018). The results also corroborate the prediction of the maintenance of looking at both the negative and the affirmative image or printed word throughout the period of time measured. However, it is notable that the subset of individuals who looked at the negative image or printed word for the counterfactual maintained their focus on the affirmative image or printed word only at a rate similar to their baseline rate, and did not increase their focus on it. The findings from the overall group analysis, and for this subset of half of the participants, support the theory that the essence of the dual meaning of a counterfactual is the comparison of reality to an imagined alternative (e.g., Byrne, 2005; Beck et al., 2006).

The differences between the two types of conditional, when they did emerge, emerged early, as Figure 2C, 5C and 7C show. They occurred at about 550 to 850 *ms* with explicit instructions to look at the objects that correspond to the meaning of what participants hear (in Experiments 1 and 3), and somewhat later without instructions (at 1700 *ms* in Experiment 2). The instruction (which may activate a controlled or top-down process) accelerates the understanding of counterfactuals in relation to the images. In particular, the differences between the two types of conditionals were due to the increase in attention to the negative image for the counterfactual. The results showed the same pattern for printed words and pictures, which demonstrates the similarities between both methodologies (McQueen and Viebahn, 2007; Primativo et al., 2016). But the tendency to focus on the negative printed word in the third experiment was perhaps even more clear-cut, as Figure 4, 6 and 8 illustrate, which is consistent with findings that visual information can impede the comprehension of negation given its symbolic nature (see Orenes and Santamaría, 2014). The findings may also have implications for the question of whether the inference of the falsity of a counterfactual’s antecedent and consequent is a “global” sentential inference accessed only at the end of the sentence (e.g., Sperber and Wilson, 1995), or a “local” sub-sentential inference accessed as soon as some trigger or cue is encountered (e.g., Levinson, 2000; see Reboul, 2004). The early processing of the negative printed word seems to suggest it is not a global inference.

Nonetheless, the data show clearly that almost half of participants did not recover the presumed facts. Why are there such striking differences between individuals in the comprehension of a counterfactual conditional? One explanation could be that they arise from some aspect of the visual world paradigm task. For example, when asked to look at the image or printed word that corresponds to the meaning of the sentence, a participant may interpret that as referring to the way things would have been in the hypothetical situation, that is, the affirmative image or printed word which corresponds to the non-actual situation that the counterfactual sentence invites one to entertain, or to what is implied about actual circumstances, that is, the negative image or printed word that corresponds to the actual circumstances as conveyed by the presupposition of the counterfactual. However, the results of Experiment 1 were replicated in Experiment 2, in which participants were not given explicit instructions to look at the image that corresponded to the meaning of the sentence, and so we can rule out the suggestion that the differences arise from differences in interpretations of the instructions.

Of course, it may also be the case that the visual world paradigm and eye-tracking provides a somewhat insensitive measure of the mental representation of counterfactuals. The objects a person fixates on need not be the only objects they are thinking about, and viewers may even use a broader attentional focus to attend to several objects (e.g., Cave and Bichot, 1999; see also Huettig and Altmann, 2005, see also Huettig and Altmann, 2011; Huettig et al., 2011b). It is also worth noting that participants rarely focused on the distractors, such as the image or printed words corresponding to “apple and strawberry” or “no apple and no strawberry.” Strictly speaking, for a counterfactual such as “if there had been oranges there would have been pears,” the distractor is also consistent with its presumed facts. For example, the image of an apple and a strawberry can be interpreted as an implicit negation of an orange and a pear (e.g., Espino and Byrne, 2018). Yet, participants focused on the image that contained an explicit negation, the orange and pear with a cross through it, or the printed words “no orange and no pear,” rather than on either of the two distractors. It may be that the explicit negation is more salient in the set of four images as corresponding to the opposite of what the counterfactual conjectured, that is, as the presumed facts. Participants may recover the presumed reality from the imagined alternative to reality conjectured in the counterfactual by negating the items mentioned. Of course, it may be more time consuming and require more cognitive steps to make the inference from “no orange and no pear” to “apple and strawberry” (e.g., Espino and Byrne, 2012; Khemlani et al., 2014; Orenes et al., 2014). Moreover, unless the context specifies a binary situation, the inference that “there is no orange and no pear” does not mean necessarily that “there is an apple and a strawberry” since there could be other fruit instead. The lack of attention to the affirmative distractor may arise because during the experimental trials the participants detected that the stories continued after the counterfactual by referring to the items in the affirmative image (e.g., orange and pear) or negative image (e.g., no orange and no pear) in the subsequent conjunction that followed the counterfactual.

An alternative potential explanation for the individual differences is that they arise from difficulties in considering different possibilities for counterfactuals. Such difficulties could arise because of differences in working memory capacity (e.g., Ferguson and Cane, 2015). Participants may focus on only one image as a consequence of limitations of working memory, given that multiple alternatives can overload processing capacity (e.g., Johnson-Laird and Byrne, 2002; Khemlani et al., 2018). Related to this proposal, the differences between individuals may reflect a failure by some participants to process the information deeply. Some participants exhibit a tendency in these sorts of tasks to construct an incomplete and shallow semantic representation (e.g., Ferreira et al., 2002; see also Erickson and Mattson, 1981; Barton and Sanford, 1993). Some participants may represent only the conjecture as a result of a heuristic “match” to what is mentioned in the conditional (e.g., Evans et al., 1999). Of course, the subjunctive mood is neither sufficient nor necessary for the communication of counterfactuality (e.g., Dudman, 1988), and some participants may tend to rely on cues of content more than linguistic mood to trigger a counterfactual interpretation of a conditional. The finding of individual differences in doing so is consistent with inference studies (Thompson and Byrne, 2002). We anticipate that more participants would envisage both the conjecture and the presumed facts for episodic counterfactuals for which the facts are known, compared to the semantic counterfactuals of the current experiments for which the facts must be presumed.

The identification of individual differences in these three experiments has consequences for the interpretation of conflicting observations in previous comprehension studies. The often-conflicting results of previous studies have been interpreted in different ways, either to support the idea that people represent only the conjecture (e.g., Evans and Over, 2004; Evans, 2007), or the idea that they represent both the conjecture and the presumed facts (e.g., Johnson-Laird and Byrne, 2002; Byrne, 2005). Even among theorists who consider that people represent both possibilities, the results have been interpreted to support the idea that the conjecture is more highly activated than the presumed facts (e.g., Nieuwland and Martin, 2012; Kulakova et al., 2013; Ferguson and Cane, 2015), or that the presumed facts are more highly activated than the conjecture (e.g., De Vega and Urrutia, 2012). It seems likely that at least some of the conflicting results reflect individual differences. Nonetheless, the data appear to rule against the theory that people only ever represent a counterfactual’s conjecture (e.g., Evans and Over, 2004; Nieuwland and Martin, 2012), since at least half of the participants in each of the experiments represented both the conjecture and the presumed facts. Instead, the data appear to show that a representation of the presumed facts (e.g., the negated conjunction) is a component of the meaning of counterfactuals compared to indicative conditionals, for those participants who reach a counter factual interpretation (e.g., Thompson and Byrne, 2002), and moreover it is very quickly available. The data also suggest, at least for those participants who envisage more than just the conjecture, that the presumed facts may be more highly activated than the conjecture (e.g., De Vega and Urrutia, 2012).

The main contribution of the present study has been to examine the online processing of counterfactual conditionals; hitherto there have been no studies to our knowledge to explore the processing of counterfactuals continuously throughout a 4000 *ms* period of time, measuring eye fixations at every 50 *ms*. Online studies that have explored counterfactuals using event-related potentials (ERP) have focused on one specific period of time (e.g., the component N400; Nieuwland, 2012) and those using eye-tracking have focused on specific intervals (e.g., Stewart et al., 2009). Other eye-tracking or ERP studies of counterfactuals have focused on the effect of the counterfactual conditional on the processing of subsequent words or sentences (e.g., Ferguson et al., 2008; Urrutia et al., 2012a). Similarly, although some studies have highlighted individual differences in the processing of counterfactuals (e.g., Ferguson and Cane, 2015), none has examined different patterns of processing in the focus on affirmative and negative images. The advantage of studying counterfactuals during a continuous 4000 *ms* period and examining fixations at every 50 *ms* is that it has revealed the important discovery that when people hear a counterfactual conditional, about half of them envisage the imagined alternative to reality only, and the other half envisage the imagined alternative to reality and the presumed facts, or the facts alone, and these representational choices occur within just the first few milliseconds after hearing the object word, e.g., “oranges,” immediately after the cue of the subjunctive mood, “would have.” We chose to time-lock our fixation measurements after the first object (e.g., “oranges”) since at that point participants can identify the target images (the ones with oranges and pears or no oranges and no pears) and differentiate them from the distractor images (the ones with apples and strawberries or no apples and no strawberries). By the time participants hear the first object word, however, they have already heard the indicative or subjunctive mood of the antecedent (if there *are*/*had been*), which may provide an additional cue about the likely mood of the consequent (notwithstanding the possibility of mixed antecedent-consequent moods). It would be useful in future studies to explore other time-locks, e.g., after “if.” A fruitful avenue for future research may also be to examine individual differences further, to identify their source, and to examine their effects not only on the comprehension of counterfactuals, but also on reasoning with counterfactuals.

## Ethics Statement

This study was carried out in accordance with the recommendations of “Comité de Ética de la Investigación y Bienestar Animal (Universidad de La Laguna)” with written informed consent from all subjects. All subjects gave written informed consent in accordance with the Declaration of Helsinki. The protocol was approved by the “Comité de Ética de la Investigación y Bienestar Animal (Universidad de La Laguna).”

## Author Contributions

IO, JG-M, and RB contributed to the design of the experiments. IO carried out the experiments and analyzed the data. IO and RB wrote the manuscript. All authors contributed to the hypotheses, interpretation of results and revised the manuscript, read and approved the submitted version.

## Conflict of Interest Statement

The authors declare that the research was conducted in the absence of any commercial or financial relationships that could be construed as a potential conflict of interest.

## Footnotes

1 As a check that participants were attending to the task, we analyzed the response accuracy and latency for these simple comprehension questions in two repeated measures analysis of variance (ANOVA) of a 2 (conditional type: indicative vs counterfactual) × 2 (conjunction type: negative vs affirmative) design. They confirmed no effects of conditional type or conjunction type, nor any interaction between the two variables, *F*<1 in every case, and the results are presented in Supplementary Table S1 in the Supplementary Material B.

2 For the record, we also carried out an analysis of fixations time-locked to the onset of the affirmative or negative conjunction, e.g., the onset of the first instance of the word “there” in “there were (no) oranges and there were (no) pears” to 3000 *ms* after that word, which was the maximum duration of the conjunctions (the average was 1.6 *ms* for affirmative and 2.2 *ms* for negative conjunctions). The analysis is consistent with the main results and for brevity and completeness we report it in the Supplementary Material B.
