# Supplementary material for: The Comprehension of Counterfactual Conditionals: Evidence From Eye-Tracking in the Visual World Paradigm
Source: Front Psychol. 2019 Jun 14;10:1172. doi: 10.3389/fpsyg.2019.01172 (PMC6587111; doi:10.3389/fpsyg.2019.01172)

Supplemental Material A: Materials

Orenes, I., Garcia-Madruga, J., Gomez-Veiga, I., Espino, O., & Byrne, R.M.J (2019). The comprehension of counterfactual conditionals: Evidence from the visual world paradigm. Frontiers in Psychology.

The conditionals and images used are presented with the English translation first and the original Spanish second. The indicative conditionals were of the form, “If there are pencils, then there are notebooks.” (Si hay lápices entonces hay libretas). The counterfactuals were of the form, “If there had been pencils, then there would have been notebooks.” (Si hubiera habido lápices entonces habría habido libretas). We provide the 36 items illustrated for indicative conditionals. Each conditional was embedded in a vignette (see text).

1. "If there are roses, then there are carnations." Si hay rosas entonces hay claveles. - "If there are daisies, then there are tulips." Si hay margaritas entonces hay tulipanes.

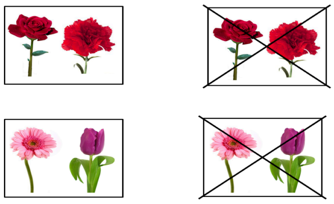

2. "If there are pencils, then there are notebooks." Si hay lápices entonces hay libretas. - "If there are sharpeners, then there are erasers." Si hay sacapuntas entonces hay gomas.

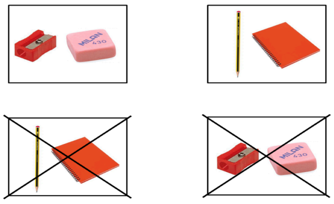

3. "If there are apples, then there are strawberries." Si hay manzanas entonces hay fresas.- "If there are oranges, then there are pears." Si hay naranjas entonces hay peras.

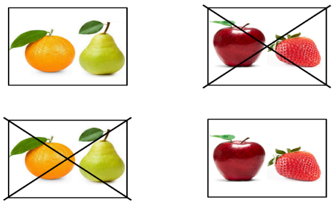

4. "If there is ham, then there is sausage." Si hay jamón entonces hay salchichón. - "If there is chorizo, then there is sobrassada." Si hay chorizo entonces hay sobrasada.

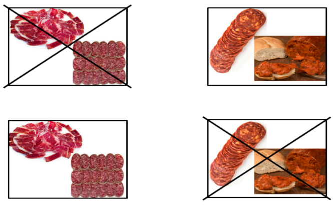

5. "If there are glasses, then there are caps." Si hay gafas entonces hay gorros. - "If there are swimsuits, then there are towels." Si hay bañadores entonces hay toallas.

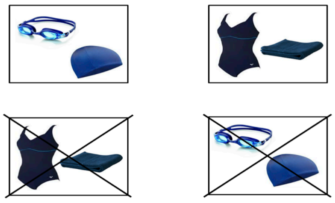

6. "If there are rabbits, then there are partridges." Si hay conejos entonces hay perdices. - "If there are boars, then there are deers." Si hay jabalíes entonces hay ciervos.

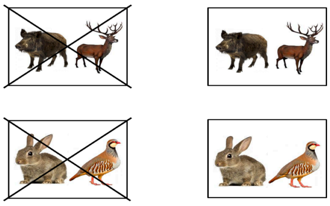

7. "If there are vans, then there are cars." Si hay furgonetas entonces hay coches. - "If there are trucks, then there are motorbikes." Si hay camiones entonces hay motos.

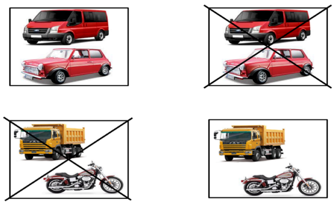

8. "If there is macaroni, then there is meat." Si hay macarrones entonces hay carne. - "If there is soup, then there is fish." Si hay sopa entonces hay pescado.

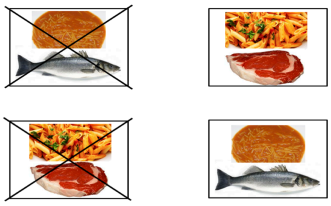

9. “If there are mice, then there are ducks.” Si hay ratones entonces hay patos. - “If there are cows, then there are cats.” Si hay vacas entonces hay gatos.

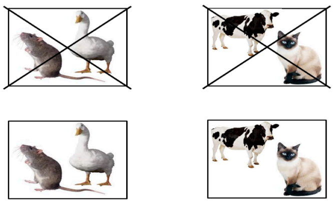

10. “If there are socks, then there are pants.” Si hay calcetines entonces hay bragas. - “If there are underpants, then there are vests.” Si hay calzoncillos entonces hay camisetas.

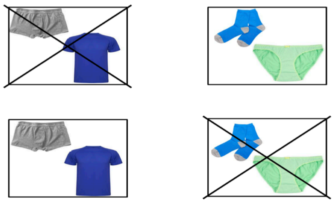

11. “If there are screws, then there are headlights.” Si hay tuercas entonces hay faros. - “If there are tires, then there are bolts.” Si hay neumáticos entonces hay tornillos.

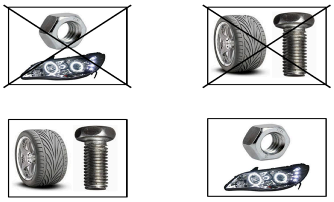

12. “If there are pies, then there are donuts.” Si hay empanadas entonces hay donuts. - “If there are pizzas, then there are croissants.” Si hay pizzas entonces hay napolitanas.

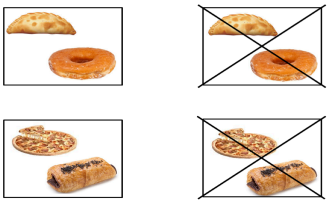

13. “If there are staples, then there are scissors.” Si hay grapas entonces hay tijeras. - “If there is glue, then there are paperclips.” Si hay pegamento entonces hay clips.

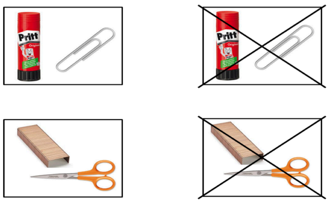

14. “If there is flour, then there are eggs.” Si hay harina entonces hay huevos. - “If there is yeast, then there is sugar.” Si hay levadura entonces hay azúcar.

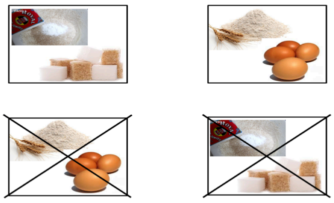

15. “If there are almonds, then there are pistachios.” Si hay almendras entonces hay pistachos. - “If there are walnuts, then there are cashews.” Si hay nueces entonces hay anacardos.

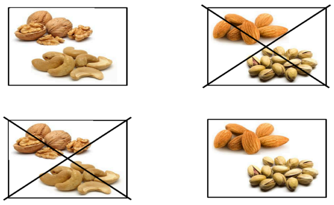

16. “If there are octopuses, then there are limpets.” Si hay pulpos entonces hay lapas. - “If there are squid, then there are crayfishes.” Si hay calamares entonces hay cangrejos.

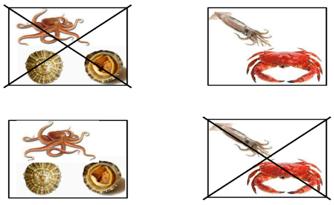

17. “If there are hammocks, then there are umbrellas.” Si hay hamacas entonces hay sombrillas. - “If there are beachbars, then there are boats.” Si hay chiringuitos entonces hay barcas.

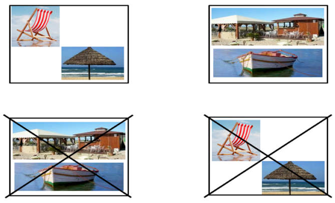

18. “If there are prawns, then there are mussels.” Si hay gambas entonces hay mejillones. - “If there is tuna, then there are clams.” Si hay atún entonces hay almejas.

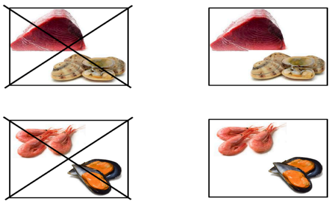

19. “If there is coffee, then there is tea.” Si hay café entonces hay te. - “If there is chocolate, then there is milk.” Si hay chocolate entonces hay leche.

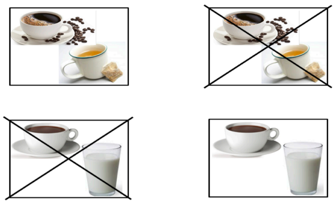

20. “If there is water, then there is beer.” Si hay agua entonces hay cerveza. - “If there is juice, then there is Pepsi.” Si hay zumo entonces hay Pepsi.

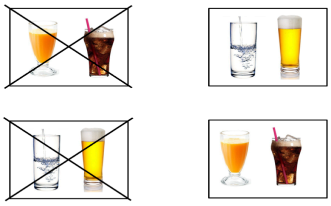

21. “If there are necklaces, then there are bracelets.” Si hay collares entonces hay pulseras. - “If there are rings, then there are earrings.” Si hay sortijas entonces hay pendientes.

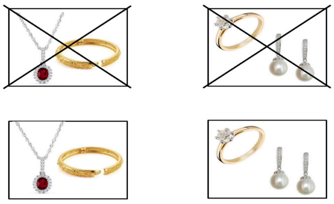

22. “If there are bags, then there are belts.” Si hay bolsos entonces hay cinturones. - “If there are scarves, then there are gloves.” Si hay bufandas entonces hay guantes.

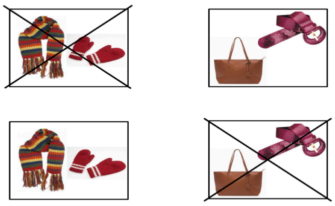

23. “If there are hospital beds, then there are thermometers.” Si hay camillas entonces hay termómetros. - “If there are bandages, then there are syringes.” Si hay vendas entonces hay jeringuillas.

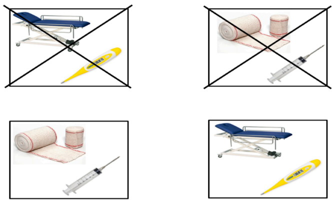

24. “If there are books, then there are markers.” Si hay libros entonces hay rotuladores. - “If there are folders, then there are pens.” Si hay carpetas entonces hay bolígrafos.

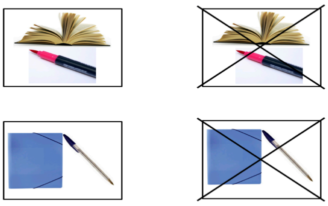

25. “If there is football, then there is basketball.” Si hay fútbol entonces hay baloncesto. - “If there is volleyball, then there is handball.” Si hay voleibol entonces hay balonmano.

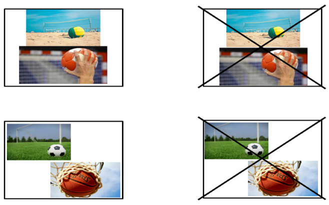

26. “If there are cards, then there are puzzles.” Si hay barajas entonces hay puzles. - “If there is Parcheesi, then there is dominos.” Si hay parchís entonces hay dominós.

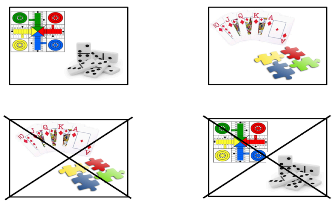

27. “If there is dance, then there is theatre.” Si hay danza entonces hay teatro. - “If there is opera, then there is flamenco.” Si hay ópera entonces hay flamenco.

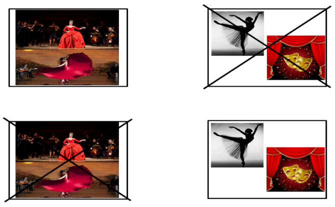

28. “If there are computers, then there are headphones.” Si hay ordenadores entonces hay auriculares. - If there are mobiles, then there are tablets.” Si hay móviles entonces hay tabletas.

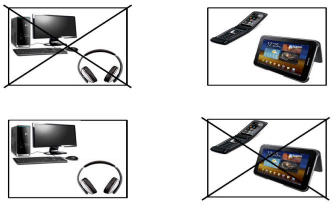

29. “If there are actors, then there are painters.” Si hay actores entonces hay pintores. - “If there are musicians, then there are writers.” Si hay músicos entonces hay escritores.

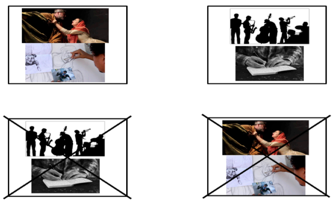

30. “If there are chickens, then there are lambs.” Si hay pollos entonces hay corderos. - “If there are pigs, then there are goats.” Si hay cerdos entonces hay cabras.

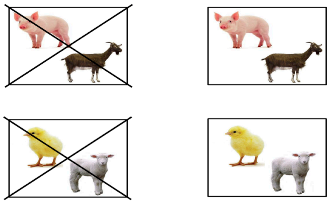

31. “If there are tables, then there are wardrobes.” Si hay mesas entonces hay armarios. - “If there are beds, then there are chairs.” Si hay camas entonces hay sillas.

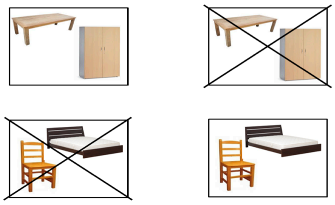

32. “If there are lentils, then there are chickpeas.” Si hay lentejas entonces hay garbanzos. - “If there are beans, then there are peas.” Si hay alubias entonces hay guisantes.

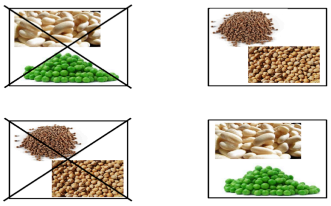

33. “If there are stamps, then there are postcards.” Si hay sellos entonces hay postales. - “If there are envelopes, then there are packages.” Si hay sobres entonces hay paquetes.

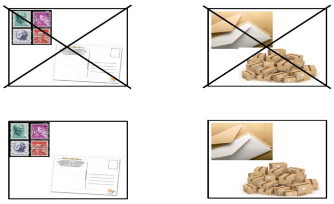

34. “If there are horses, then there are pigeons.” Si hay caballos entonces hay palomas. - “If there are dogs, then there are chimpanzees.” Si hay perros entonces hay chimpancés.

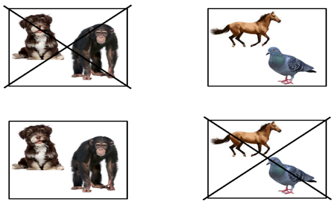

35. “If there are litterbins, then there are streetlights.” Si hay papeleras entonces hay farolas. - “If there are flowers, then there are fountains.” Si hay flores entonces hay fuentes.

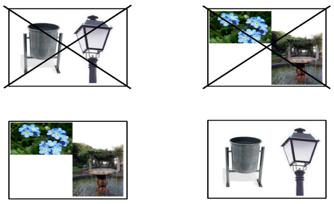

36. “If there is lettuce, then there are onions.” Si hay lechugas entonces hay cebollas. - “If there are tomatoes, then there are cucumbers.” Si hay tomates entonces hay pepinos.

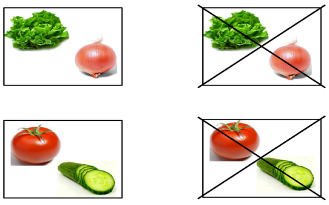

Supplement: Supplementary file 1 [file Data_Sheet_1.PDF]
