# Supplementary material for: The Comprehension of Counterfactual Conditionals: Evidence From Eye-Tracking in the Visual World Paradigm
Source: Front Psychol. 2019 Jun 14;10:1172. doi: 10.3389/fpsyg.2019.01172 (PMC6587111; doi:10.3389/fpsyg.2019.01172)
Supplement: Supplementary file 2 [file Data_Sheet_2.pdf]

**Supplemental Material B: Additional Statistical Analyses**

*Orenes, I., Garcia-Madruga, J., Gomez-Veiga, I., Espino, O., & Byrne, R.M.J (2019). The comprehension of counterfactual conditionals: Evidence from the visual world paradigm. Frontiers in Psychology.*

Table S1. *The accuracy and latency to read and respond to the simple comprehension sentences in Experiments 1, 2 and 3.*

| | | Accuracy | Latency |
|---|---|---|---|
| Experiment 1 | | | |
| Counterfactual | Affirmative Conjunction | 8.2 (0.99) | 1621.09 (250.46) |
| | Negative Conjunction | 7.9 (1.06) | 1722.84 (349.67) |
| Indicative | Affirmative Conjunction | 8.3 (0.81) | 1704.03 (367.16) |
| | Negative Conjunction | 8.0 (1.08) | 1689.60 (285.23) |
| Experiment 2 | | | |
| Counterfactual | Affirmative Conjunction | 8.33 (0.84) | 1892.19 (480.99) |
| | Negative Conjunction | 8.22 (1.01) | 1754.11 (318.12) |
| Indicative | Affirmative Conjunction | 8.44 (0.86) | 1895.49 (437.83) |
| | Negative Conjunction | 8.50 (0.62) | 1850.44 (382.44) |
| Experiment 3 | | | |
| Counterfactual | Affirmative Conjunction | 8.05 (1.09) | 1801.98 (348.35) |
| | Negative Conjunction | 8.45 (0.86) | 1781.52 (396.12) |
| Indicative | Affirmative Conjunction | 8.45 (0.86) | 1892.93 (487.71) |
| | Negative Conjunction | 8.09 (1.06) | 1844.35 (377.85) |

**Additional statistical analyses for Experiment 1**

*Affirmative and negative conjunctions t-tests against baseline.*

For affirmative conjunctions, no change was observed in fixation for indicative conditionals on the affirmative images or negative images; participants increased fixation for counterfactuals on the affirmative image from 1150 *ms* (*pFDRcorr* = 0.029), and decreased on the negative image (*pFDRcorr* = 0.005). For negative conjunctions, they increase their fixations for the indicative conditionals on the negative image from 900 *ms* (*pFDRcorr* = 0.003) and decrease on the affirmative image from 850 *ms* (*pFDRcorr* = 0.037). They increase their fixations for the counterfactual conditionals from 600 *ms* on the negative image (*pFDRcorr* = 0.005) and decrease on the affirmative image from 850 *ms* (*pFDRcorr* = 0.027), as Figure S1 shows.

Figure S1. *Probabilities of fixations for indicative conditionals for the affirmative conjunction (A) and the negative conjunction (B), and for counterfactual conditionals for the affirmative conjunction (C) and the negative conjunction (D) in Experiment 1. Error bars are 95% confidence intervals within participants.*

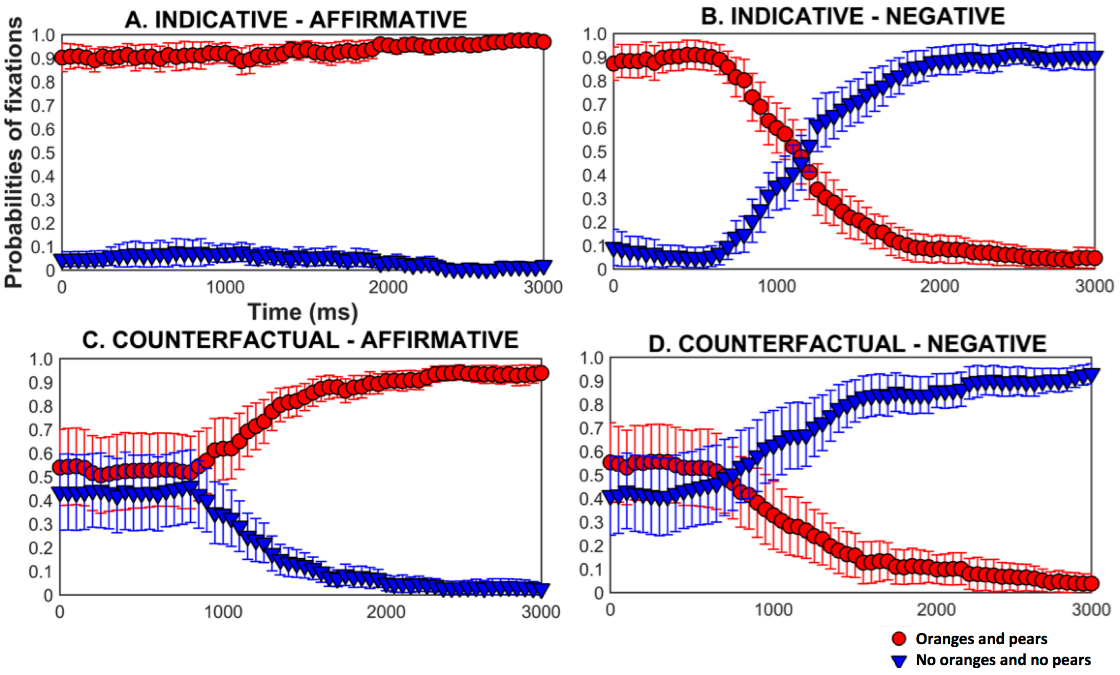

*T-tests against baseline, for the distractor images (analysis by participants)*

For the indicative conditional fixations on the distractors decreased for the affirmative distractor (from 550 *ms*, *pFDR-corr* = 0.015), and the negative distractor (from 650 *ms*, *pFDR-corr* = 0.030). For the counterfactual fixations on the distractors decreased, for the affirmative distractor (from 400 *ms*, *pFDR-corr* = 0.038) and the negative distractor (from 500 *ms*, *pFDR-corr* = 0.021).

*Growth curve analysis*

Growth curve analysis (Mirman, 2014) was used to analyze the time course of fixation from 300 *ms* to 2000 *ms* after target word onset (from the earliest word-driven fixations to when target fixation had plateaued). The overall time course of target fixations was captured with a third-order (cubic) orthogonal polynomial terms and fixed effects of condition (indicative vs. counterfactual, within participants) on all time terms. The

Table S2. *Parameter estimates for analysis of effect of conditional on affirmative and negative image in Experiment 1.*

| Image | Estimate | Std.Error | t.value | p |
|---|---|---|---|---|
| ***Affirmative Image*** | | | | |
| Intercept | 0.540 | 0.037 | 14.483 | <0.001 |
| Linear | 0.142 | 0.085 | 1.672 | 0.094 |
| Quadratic | -0.184 | 0.039 | -4.695 | <0.001 |
| Cubic | 0.043 | 0.034 | 1.239 | 0.215 |
| ConditionIndicative | 0.188 | 0.047 | 4.020 | <0.001 |
| Linear: ConditionIndicative | 0.649 | 0.120 | 5.424 | <0.001 |
| Quadratic: ConditionIndicative | -0.118 | 0.055 | -2.162 | 0.031 |
| Cubic: ConditionIndicative | 0.037 | 0.048 | 0.768 | 0.442 |
| ***Negative Image*** | | | | |
| Intercept | 0.293 | 0.038 | 7.760 | <0.001 |
| Linear | 0.331 | 0.086 | 3.860 | <0.001 |
| Quadratic | -0.044 | 0.034 | -1.255 | 0.209 |
| Cubic | 0.029 | 0.029 | 1.005 | 0.315 |
| ConditionIndicative | -0.217 | 0.041 | -5.348 | <0.001 |
| Linear: ConditionIndicative | -0.534 | 0.121 | -4.409 | <0.001 |
| Quadratic: ConditionIndicative | 0.045 | 0.048 | 0.925 | 0.355 |
| Cubic: ConditionIndicative | 0.017 | 0.035 | 0.475 | 0.635 |

model also included participant and participant-by-condition random effects on all time terms. The indicative condition was treated as the reference (baseline) and relative parameters estimated for the counterfactual condition. Statistical significance (p-values) for individual parameter estimates was assessed using the normal approximation (treating the t-value as a z-value). All analyses were carried out in R version 3.5.3 using the lme4 package.

Table S2 shows the parameter estimates and their standard errors along with p-values estimated using the normal approximation for the t-value for affirmative and negative images. For the affirmative image, there was a significant effect of conditional on the intercept (*Estimate* = 0.188, *SE* = 0.047, $p < 0.001$), indicating overall higher fixation probability for the indicative conditional than the counterfactual conditional (a condition effect on the intercept – the indicative condition had a statistically significant positive estimate). There was also an effect of conditional on the slope (*Estimate* = 0.649, *SE* = 0.120, $p < 0.001$), indicating a steeper slope for the indicative than counterfactual conditional, and the quadratic (*Estimate* = -0.118, *SE* = 0.055, $p = 0.031$), indicating that the indicative and counterfactual conditionals differed in the processing. For the negative image, we found the opposite pattern. There was a significant effect of conditional on the intercept (*Estimate* = -0.217, *SE* = 0.041, $p < 0.001$), indicating overall *lower* fixation probability for the indicative conditional than the counterfactual conditional, and slope (*Estimate* = -0.534, *SE* = 0.121, $p < 0.001$), indicating a steeper slope for the counterfactual than the indicative conditional. In sum, this analysis shows that the comprehension of both types of conditionals is different in the intercept and the slope. People looked at the affirmative image more for the indicative than the counterfactual, and they looked at the negative image more for the counterfactual than the indicative conditional. The slope or increase of fixations on the affirmative image was faster for the indicative than the counterfactual conditional, and the opposite was the case for the negative image.

*T-tests against baseline, for the distractor images (analysis by items)*

For the indicative conditional fixations on the distractors decreased, for the affirmative distractor (from 550 *ms*, *pFDR-corr* = 0.008), and the negative distractor (from 550 *ms*,

*pFDR-corr* = 0.034). For the counterfactual fixations on the distractor images decreased, for the affirmative distractor (from 250 *ms*, *pFDR-corr* = 0.023) and the negative distractor (from 500 *ms*, *pFDR-corr* = 0.019).

*T-tests against baseline, for the distractor images (Individual differences analysis)*

For the indicative conditional fixations on the distractor images decreased, for the affirmative distractor (group 1 from 550 *ms*, *pFDR-corr* = 0.032; group 2 from 650 *ms*, *pFDR-corr* = 0.041), and the negative distractor (group 1, no change; group 2 from 300 *ms*, *pFDR-corr* = 0.039). For the counterfactual fixations on the distractor images decreased for the affirmative distractor (group 1 from 600 *ms, pFDR-corr* = 0.035; group 2 from 900 *ms, pFDR-corr* = 0.033), and for the negative distractor (group 1, no change; group 2, from 300 *ms*, *pFDR-corr* = 0.013).

**Additional statistical analyses for Experiment 2**

*Affirmative and negative conjunctions t-tests against baseline.*

For affirmative conjunctions, participants increased their focus on the affirmative image from 1100 *ms* for indicative conditionals (*pFDR-corr* = 0.029) and counterfactual conditionals (*pFDR-corr* = 0.009). For counterfactuals there was a decrease in fixation on the negative image from 1500 *ms* (*pFDR-corr* = 0.006), no change was observed for indicative conditionals as the negative image had not been fixated on previously, as Figure S2 shows. For negative conjunctions, participants increased their fixations on the negative image (*pFDR-corr* = 0.007) and decreased their fixations on the affirmative image (*pFDR-corr* = 0.031) from 850 *ms* for indicative conditionals. For counterfactuals, participants increased their fixation on the negative image from 950 *ms* (*pFDR-corr* = 0.031) and decreased on the affirmative image from 1050 *ms* (*pFDR-corr* = 0.014), as Figure S2 shows.

Figure S2. *Probabilities of fixations for indicative conditionals for the affirmative conjunction (A) and the negative conjunction (B), and for counterfactual conditionals for the affirmative conjunction (C) and the negative conjunction (D) in Experiment 2. Error bars are 95% confidence intervals within participants.*

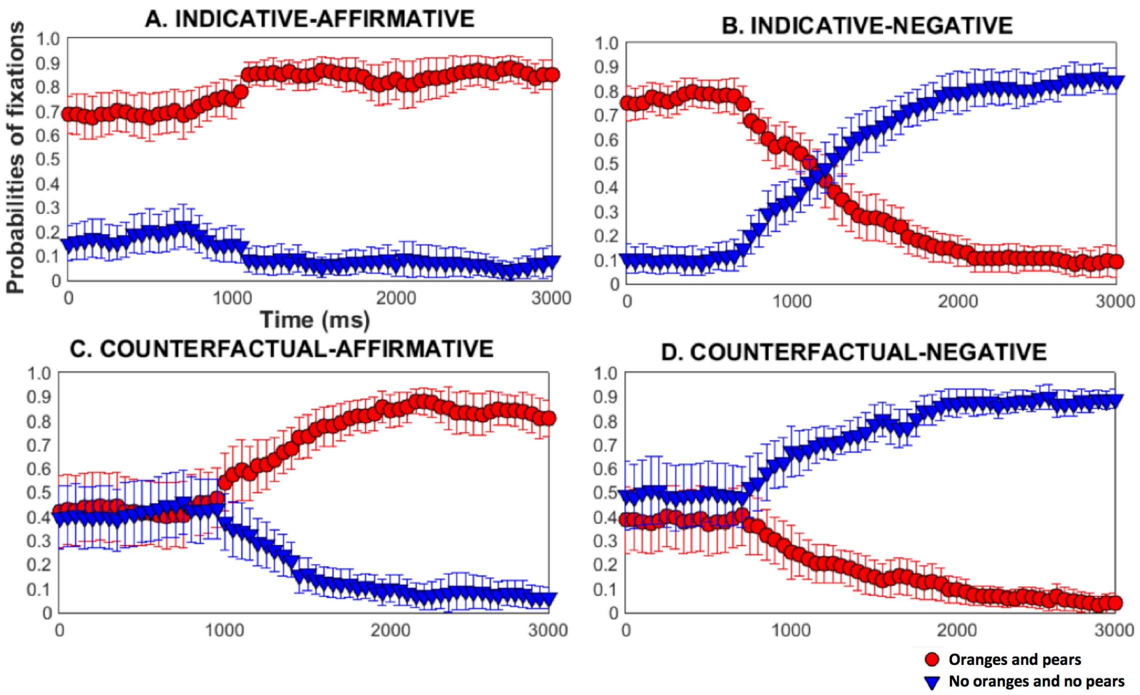

*T-tests against baseline, for the distractor images (analysis by participants)*

For the indicative conditional fixations on the distractors decreased, for the affirmative distractor (from 500 *ms*, *pFDR-corr* = 0.036), and for the negative distractor (from 750 *ms*, *pFDR-corr* = 0.030). For the counterfactual there was a decrease in fixations on the affirmative distractor (from 450 *ms*, *pFDR-corr* = 0.012), and for the negative distractor (from 700 *ms*, *pFDR-corr* = 0.032).

*Growth curve analysis*

The results for the growth curve analysis are presented in Table S3 which shows the parameter estimates and their standard errors along with p-values estimated using the normal approximation for the t-value for affirmative and negative images. For the affirmative image, there was a marginal effect of conditional on the intercept (*Estimate* = 0.090, *SE* = 0.047, *p* = 0.059), indicating overall higher fixation probability for the indicative

Table S3. *Parameter estimates for analysis of effect of conditional on affirmative and negative image in Experiment 2.*

| Image | Estimate | Std.Error | t.value | p |
|---|---|---|---|---|
| **Affirmative image** | | | | |
| Intercept | 0.557 | 0.041 | 13.589 | <0.001 |
| Linear | 0.306 | 0.104 | 2.937 | 0.003 |
| Quadratic | -0.262 | 0.062 | -4.183 | <0.001 |
| Cubic | 0.003 | 0.036 | 0.071 | 0.943 |
| ConditionIndicative | 0.090 | 0.047 | 1.888 | 0.059 |
| Linear: ConditionIndicative | 0.350 | 0.144 | 2.424 | 0.015 |
| Quadratic: ConditionIndicative | 0.060 | 0.083 | 0.723 | 0.469 |
| Cubic: ConditionIndicative | 0.019 | 0.043 | 0.447 | 0.654 |
| **Negative image** | | | | |
| Intercept | 0.220 | 0.030 | 7.363 | <0.001 |
| Linear | 0.179 | 0.075 | 2.379 | 0.017 |
| Quadratic | 0.009 | 0.036 | 0.255 | 0.799 |
| Cubic | 0.045 | 0.023 | 1.995 | 0.046 |
| ConditionIndicative | -0.084 | 0.038 | -2.243 | 0.025 |
| Linear: ConditionIndicative | -0.298 | 0.094 | -3.189 | 0.001 |
| Quadratic: ConditionIndicative | -0.089 | 0.046 | -1.923 | 0.055 |
| Cubic: ConditionIndicative | -0.031 | 0.030 | -1.036 | 0.300 |

conditional than the counterfactual (based on an analysis of the time course of fixation from 300 *ms* to 2000 *ms*). There was also an effect of conditional on the slope (*Estimate* = 0.350, *SE* = 0.144, $p < 0.015$), indicating a steeper slope for the indicative than counterfactual conditional. For the negative image, we found the opposite pattern. There was a significant effect of conditional on the intercept (*Estimate* = -0.084, *SE* = 0.038, $p$ = 0.025), indicating overall *lower* fixation probability for the indicative conditional than the counterfactual; and slope (*Estimate* = -0.298, *SE* = 0.094, $p = 0.001$), indicating a steeper slope for the counterfactual compared to the indicative conditional.

*T-tests against baseline, for the distractor images (Individual differences analysis)*

For the indicative conditional fixations decreased for the affirmative distractor (group 1, from 450 *ms*, *pFDR-corr* = 0.033; group 2 from 1000 *ms*, *pFDR-corr* = 0.024), and for the negative distractor (group 1, from 1000 *ms*, *pFDR-corr* = 0.033; group 2 from 750 *ms* (*pFDR-corr* = 0.041). For the counterfactual fixations decreased for the affirmative distractor (group 1 from 450 *ms pFDR-corr* = 0.025; group 2 from 900 *ms*, *pFDR-corr* = 0.028), and the negative distractor (group 1 from 750 *ms pFDR-corr* = 0.040; group 2, no significant change).

## Additional statistical analyses for Experiment 3

*Affirmative and negative conjunctions t-tests against baseline.*

For affirmative conjunctions, no change was observed in fixation for indicative conditionals on the affirmative words (the same as Experiment 1), or negative words; participants increased fixation for counterfactuals on the affirmative word (*pFDRcorr* = 0.033), and decreased on the negative word (*pFDRcorr* = 0.025) from 950 *ms*. For negative conjunctions, they increased their fixations for the indicative conditionals on the negative word (*pFDRcorr* = 0.007) and decreased on the affirmative word (*pFDRcorr* = 0.023) from 800 *ms*. They increased their fixations for the counterfactual conditionals on the negative word (*pFDRcorr* = 0.019) and decreased on the affirmative word (*pFDRcorr* = 0.009) from 850 *ms*, as Figure S3 shows.

Figure S3. *Probabilities of fixations for indicative conditionals for the affirmative conjunction (A) and the negative conjunction (B), and for counterfactual conditionals for the affirmative conjunction (C) and the negative conjunction (D) in Experiment 3. Error bars are 95% confidence intervals within participants.*

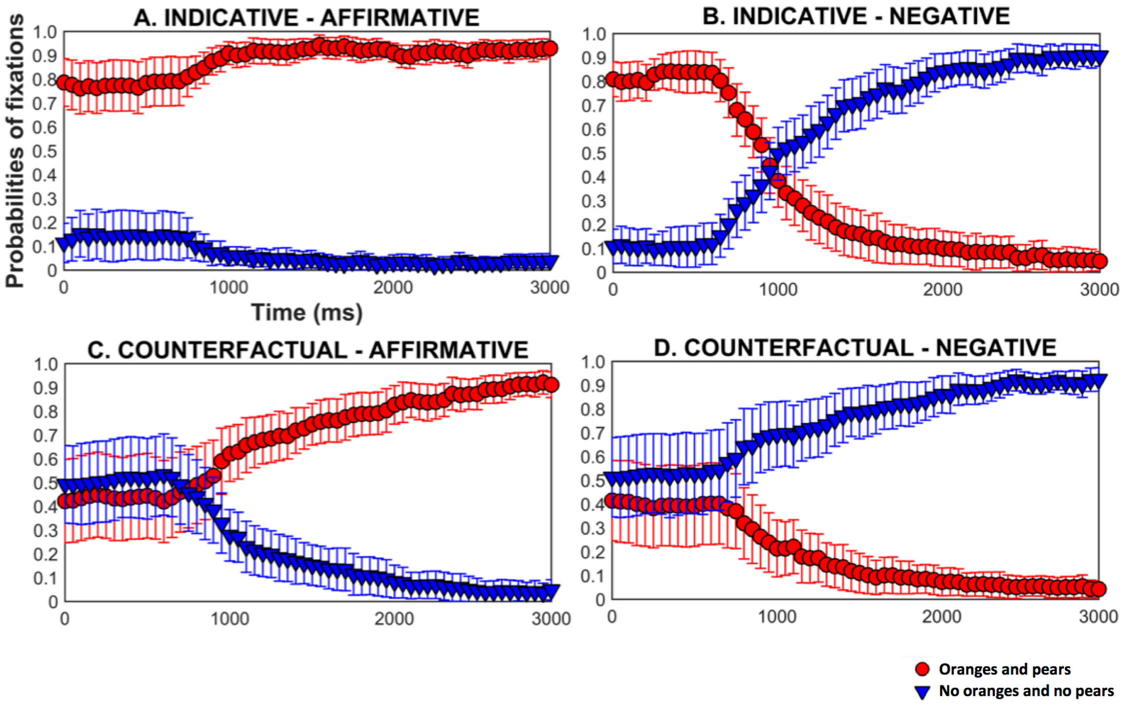

*T-tests against baseline, for the distractor words (analysis by participants)*

For the indicative conditional fixations on the distractors decreased, for the affirmative distractor (from 650 *ms, pFDR-corr* = 0.034), and for the negative distractor (from 500 *ms*, *pFDR-corr* = 0.042). For the counterfactual fixations on the distractors decreased, for the affirmative distractor (from 400 *ms*, *pFDR-corr* = 0.019), and for the negative distractor (from 450 *ms*, *pFDR-corr* = 0.014).

*Growth curve analysis*

The results for the growth curve analysis are presented in Table S4 which shows the parameter estimates and their standard errors along with p-values estimated using the

Table S4. *Parameter estimates for analysis of effect of conditional on affirmative and negative printed words in Experiment 3.*

| Image | Estimate | Std.Error | t.value | p |
|---|---|---|---|---|
| **Affirmative word** | | | | |
| Intercept | 0.400 | 0.044 | 9.014 | <0.001 |
| Linear | 0.056 | 0.109 | 0.511 | 0.609 |
| Quadratic | -0.034 | 0.040 | -0.846 | 0.397 |
| Cubic | 0.077 | 0.038 | 2.047 | 0.041 |
| ConditionIndicative | 0.245 | 0.049 | 5.032 | <0.001 |
| Linear: ConditionIndicative | 0.690 | 0.145 | 4.746 | <0.001 |
| Quadratic: ConditionIndicative | -0.247 | 0.045 | -5.444 | <0.001 |
| Cubic: ConditionIndicative | 0.015 | 0.052 | 0.290 | 0.772 |
| **Negative word** | | | | |
| Intercept | 0.411 | 0.041 | 9.977 | <0.001 |
| Linear | 0.249 | 0.096 | 2.588 | 0.009 |
| Quadratic | -0.123 | 0.044 | -2.796 | 0.005 |
| Cubic | 0.053 | 0.031 | 1.702 | 0.089 |
| ConditionIndicative | -0.267 | 0.052 | -5.184 | <0.001 |
| Linear: ConditionIndicative | -0.489 | 0.120 | -4.071 | <0.001 |
| Quadratic: ConditionIndicative | 0.193 | 0.061 | 3.180 | 0.001 |
| Cubic: ConditionIndicative | -0.041 | 0.036 | -1.144 | 0.253 |

normal approximation for the t-value for affirmative and negative printed words. For the affirmative words, there was a significant effect of conditional on the intercept

(*Estimate* = 0.245, *SE* = 0.049, $p < 0.001$), indicating overall higher fixation probability for the indicative conditional than the counterfactual; the slope (*Estimate* = 0.690, *SE* = 0.145, $p < 0.001$), indicating a steeper slope for the indicative than the counterfactual; and the quadratic (*Estimate* = -0.247, *SE* = 0.045, $p < 0.001$), indicating that the indicative and counterfactual conditionals differed in the processing. For the negative words, we found the opposite pattern. There was a significant effect of conditional on the intercept (*Estimate* = -0.267, *SE* = 0.052, $p < 0.001$), indicating overall *lower* fixation probability for the indicative conditional than the counterfactual; the slope (*Estimate* = -0.534, *SE* = 0.121, $p < 0.001$), indicating a steeper slope for the counterfactual than the indicative; and the quadratic (*Estimate* = 0.193, *SE* = 0.061, $p$ = 0.001), indicating that the indicative and counterfactual conditionals differed in the processing.

*T-tests against baseline, for the distractor words (Individual differences analysis)*

Individual differences analysis: For the indicative conditional fixations decreased for the affirmative distractor (group 1 from 700 *ms*, *pFDR-corr* = 0.028; group 2 from 800 *ms*, *pFDR-corr* = 0.038), and for the negative distractor (group 1 from 150 *ms*, *pFDR-corr* = 0.029; group 2 from 750 *ms* (*pFDR-corr* = 0.039). For the counterfactual fixations decreased for the affirmative distractor (group 1, no significant change; group 2 from 300 *ms*, *pFDR-corr* = 0.022) and for the negative distractor (group 1, from 650 *ms* *pFDR-corr* = 0.034; group 2 from 500 *ms* *pFDR-corr* = 0.027).

**Analyses for the three experiments combined**

*T-tests against baseline, for the distractor images (Individual differences analysis for the three experiments combined)*

For the indicative conditional fixations decreased for the affirmative distractor (group 1 from 500 *ms*, *pFDR-corr* = 0.019; group 2, from 600 *ms*, *pFDR-corr* = 0.038), and for the negative distractor (group 1 from 450 *ms*, *pFDR-corr* = 0.036; group 2, from 550 *ms*, *pFDR-corr* = 0.030). For the counterfactual fixations decreased for the affirmative distractor (group 1 from 450 *ms*, *pFDR-corr* = 0.013; group 2 from 300 *ms*, *pFDR-corr* = 0.006), and the negative distractor (group 1 from 200 *ms*, *pFDR-corr* = 0.039; group 2 from 500 *ms* (*pFDR-corr* = 0.026).

*Growth curve analysis for the three experiments combined*

We carried out a growth curve analysis of the combined data from the three experiments. Table S5 shows the parameter estimates and their standard errors along with p-values estimated using the normal approximation for the t-value for affirmative and negative images or printed words. For group 2, for the affirmative image or printed words, there was a significant effect of conditional on the intercept (*Estimate* = 0.334, *SE* = 0.036, $p < 0.001$), indicating overall higher fixation probability for the indicative conditional than the counterfactual; the slope (*Estimate* = 1.070, *SE* = 0.078, $p < 0.001$), indicating a steeper slope for the indicative than the counterfactual; and the quadratic (*Estimate* = -0.163, *SE* = 0.050, $p = 0.001$), indicating that the indicative and counterfactual conditionals differed in the processing. For the negative image or printed words, we found the opposite pattern. There was a significant effect of conditional on the intercept (*Estimate* = -0.345, *SE* = 0.034, $p < 0.001$), indicating overall *lower* fixation probability for the indicative conditional than the counterfactual; the slope (*Estimate* = -0.873, *SE* = 0.066, $p < 0.001$), indicating a steeper slope for the counterfactual than the indicative; and the quadratic (*Estimate* = 0.126, *SE* = 0.051, $p = 0.014$), indicating that the indicative and counterfactual conditionals differed in the processing.

Table S5. *Parameter estimates for analysis of effect of conditional on affirmative and negative image or printed words in Experiments 1-3.*

| | Group 1 | | | | Group 2 | | | |
|---|---|---|---|---|---|---|---|---|
| Image | Estimate | Std.Error | t.value | p | Estimate | Std.Error | t.value | p |
| **Affirmative image/word** | | | | | | | | |
| Intercept | 0.686 | 0.027 | 25.753 | <0.001 | 0.318 | 0.027 | 11.727 | <0.001 |
| Linear | 0.623 | 0.057 | 10.921 | <0.001 | -0.274 | 0.059 | -4.618 | <0.001 |
| Quadratic | -0.176 | 0.047 | -3.733 | <0.001 | -0.111 | 0.037 | -3.032 | 0.002 |
| Cubic | 0.006 | 0.028 | 0.219 | 0.826 | 0.069 | 0.032 | 2.164 | 0.030 |
| ConditionIndicative | 0.007 | 0.020 | 0.336 | 0.737 | 0.334 | 0.036 | 9.150 | <0.001 |
| Linear: ConditionIndicative | 0.052 | 0.079 | 0.657 | 0.511 | 1.070 | 0.078 | 13.673 | <0.001 |
| Quadratic: ConditionIndicative | -0.083 | 0.059 | -1.409 | 0.159 | -0.163 | 0.050 | -3.251 | 0.001 |
| Cubic: ConditionIndicative | 0.041 | 0.033 | 1.220 | 0.222 | 0.017 | 0.044 | 0.379 | 0.705 |
| **Negative image/word** | | | | | | | | |
| Intercept | 0.114 | 0.015 | 7.502 | <0.001 | 0.486 | 0.026 | 18.562 | <0.001 |
| Linear | -0.203 | 0.042 | -4.823 | <0.001 | 0.644 | 0.051 | 12.65 | <0.001 |
| Quadratic | -0.010 | 0.028 | -0.361 | 0.718 | -0.112 | 0.037 | -3.02 | 0.002 |
| Cubic | 0.042 | 0.020 | 2.089 | 0.037 | 0.048 | 0.026 | 1.856 | 0.063 |
| ConditionIndicative | -0.018 | 0.012 | -1.477 | 0.140 | -0.345 | 0.034 | -10.04 | <0.001 |
| Linear: ConditionIndicative | 0.057 | 0.049 | 1.176 | 0.239 | -0.873 | 0.066 | -13.15 | <0.001 |
| Quadratic: ConditionIndicative | -0.004 | 0.039 | -0.115 | 0.908 | 0.126 | 0.051 | 2.455 | 0.014 |
| Cubic: ConditionIndicative | -0.015 | 0.022 | -0.672 | 0.501 | -0.024 | 0.033 | -0.718 | 0.473 |
