# Supplementary material for: The Comprehension of Counterfactual Conditionals: Evidence From Eye-Tracking in the Visual World Paradigm
Source: Front Psychol. 2019 Jun 14;10:1172. doi: 10.3389/fpsyg.2019.01172 (PMC6587111; doi:10.3389/fpsyg.2019.01172)
Supplement: Supplementary file 3 [file Data_Sheet_3.pdf]

Supplemental Material C: Individual differences

Orenes, I., Garcia-Madruga, J., Gomez-Veiga, I., Espino, O., & Byrne, R.M.J (2019). The comprehension of counterfactual conditionals: Evidence from the visual world paradigm. Frontiers in Psychology.

Probabilities of fixations for counterfactuals are presented for each individual participant in each of the three experiments.

Experiment 1

Probabilities of fixations for counterfactuals are presented for each individual participant in Experiment 1. The 11 participants who looked at the affirmative image only are presented first, followed by the 7 participants who looked at the negative image only, and then the 5 participants who looked at both images.

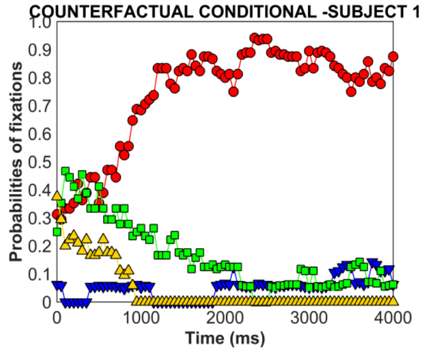

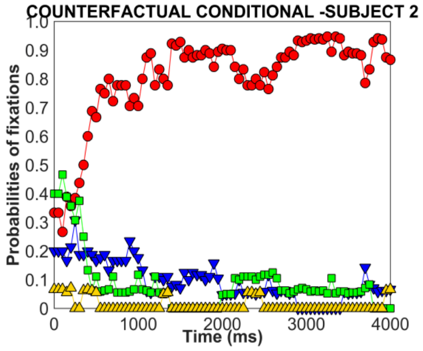

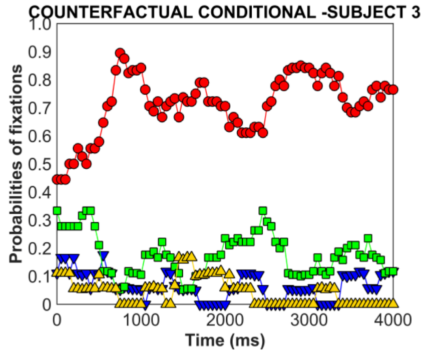

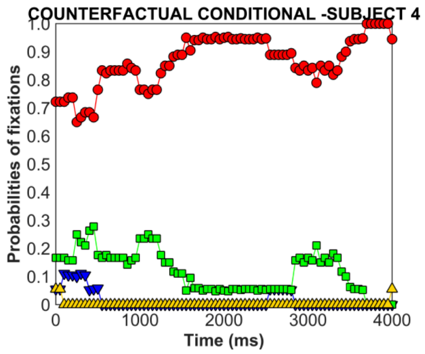

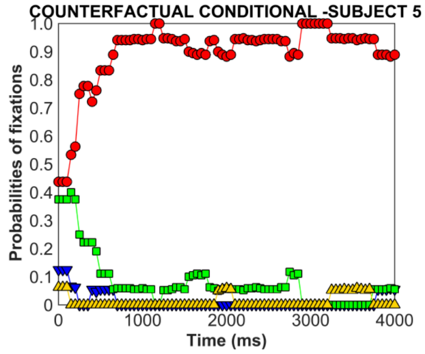

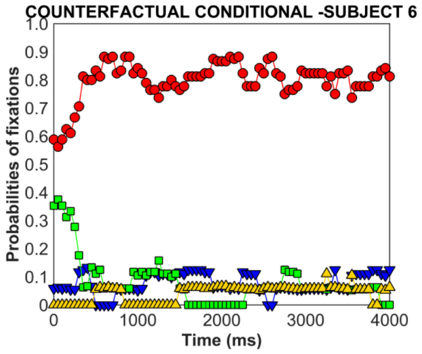

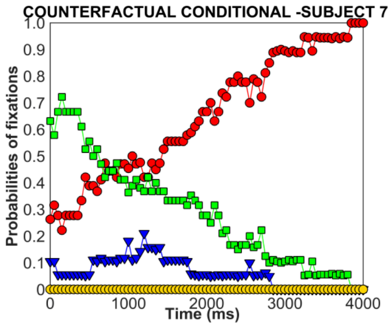
COUNTERFACTUAL CONDITIONAL -SUBJECT 7
Probabilities of fixations
Time (ms)

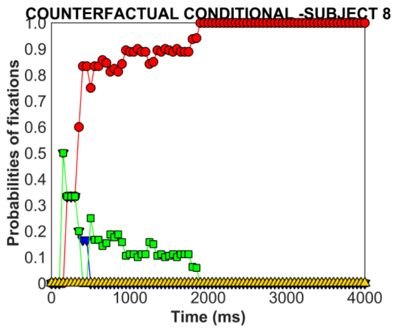
COUNTERFACTUAL CONDITIONAL -SUBJECT 8
Probabilities of fixations
Time (ms)

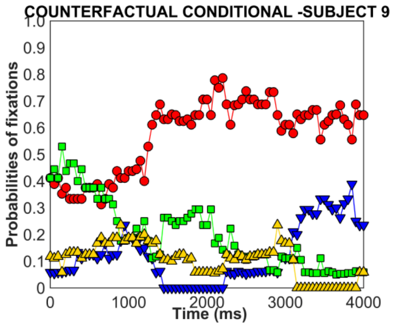
COUNTERFACTUAL CONDITIONAL -SUBJECT 9
Probabilities of fixations
Time (ms)

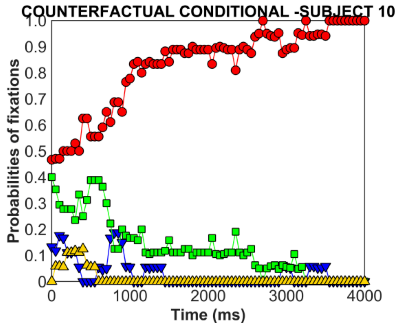
COUNTERFACTUAL CONDITIONAL -SUBJECT 10
Probabilities of fixations
Time (ms)

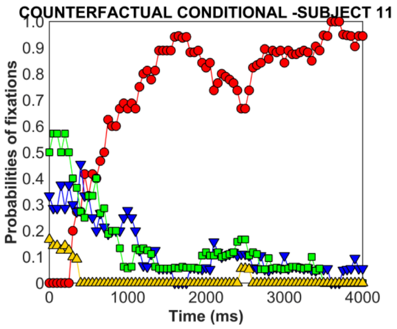
COUNTERFACTUAL CONDITIONAL -SUBJECT 11
Probabilities of fixations
Time (ms)

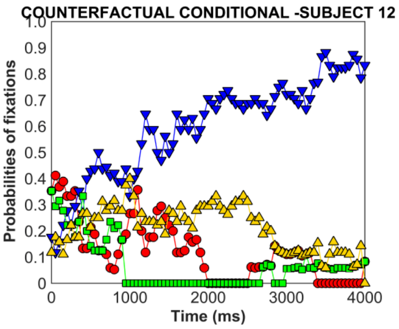
COUNTERFACTUAL CONDITIONAL -SUBJECT 12
Probabilities of fixations
Time (ms)

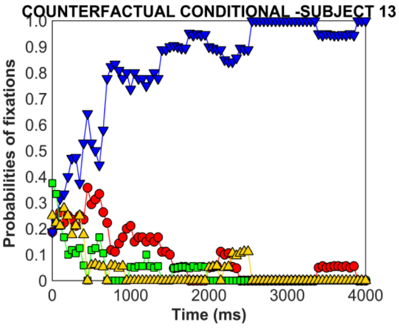
COUNTERFACTUAL CONDITIONAL -SUBJECT 13
Probabilities of fixations
Time (ms)

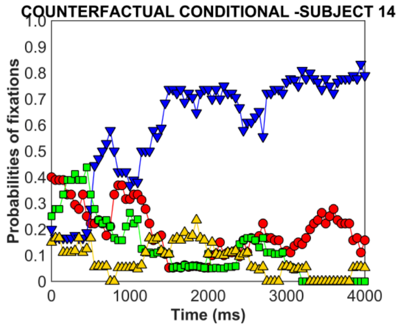
COUNTERFACTUAL CONDITIONAL -SUBJECT 14
Probabilities of fixations
Time (ms)

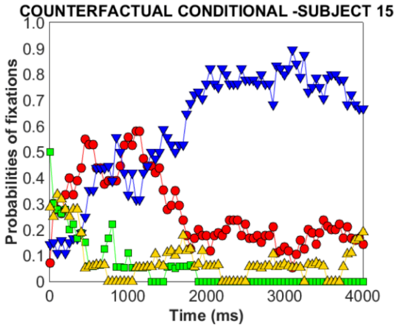
COUNTERFACTUAL CONDITIONAL -SUBJECT 15
Probabilities of fixations
Time (ms)

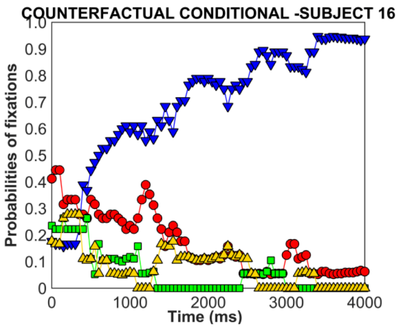
COUNTERFACTUAL CONDITIONAL -SUBJECT 16
Probabilities of fixations
Time (ms)

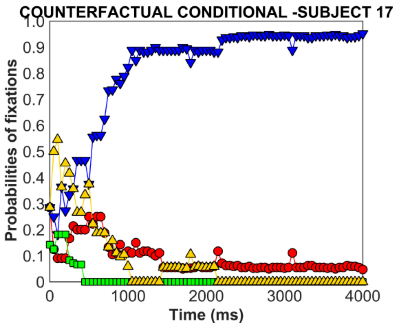
COUNTERFACTUAL CONDITIONAL -SUBJECT 17
Probabilities of fixations
Time (ms)

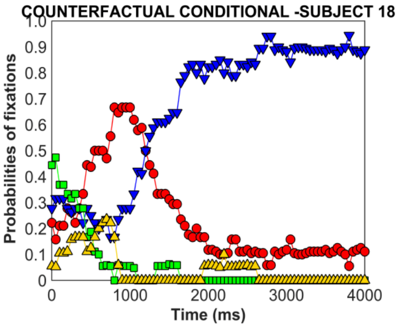
COUNTERFACTUAL CONDITIONAL -SUBJECT 18
Probabilities of fixations
Time (ms)

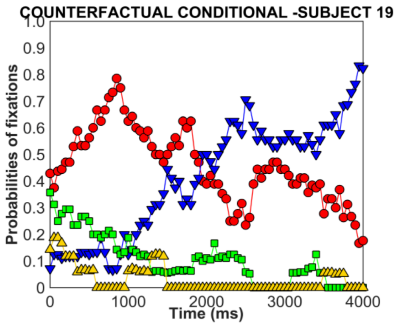
COUNTERFACTUAL CONDITIONAL -SUBJECT 19
Probabilities of fixations
Time (ms)

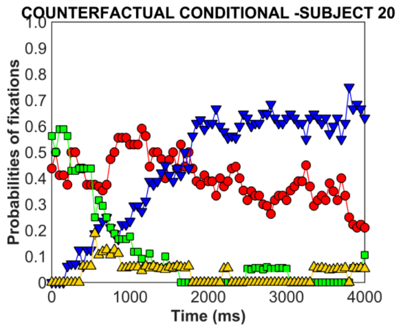
COUNTERFACTUAL CONDITIONAL -SUBJECT 20
Probabilities of fixations
Time (ms)

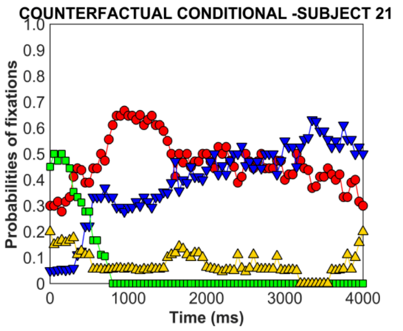
COUNTERFACTUAL CONDITIONAL -SUBJECT 21
Probabilities of fixations
Time (ms)

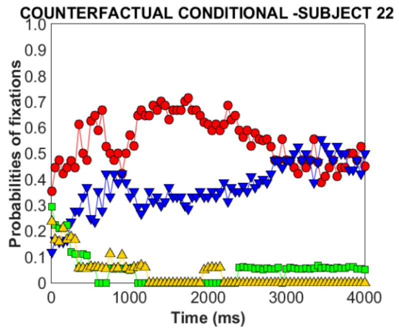
COUNTERFACTUAL CONDITIONAL -SUBJECT 22
Probabilities of fixations
Time (ms)

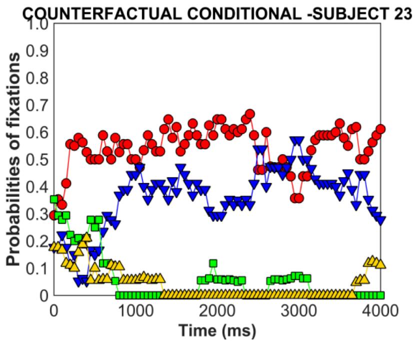
COUNTERFACTUAL CONDITIONAL -SUBJECT 23
Probabilities of fixations
Time (ms)

Experiment 2

Probabilities of fixations for counterfactuals are presented for each individual participant in Experiment 2. The 10 participants who looked at the affirmative image only are presented first, then the 4 participants who looked at the negative image only, and then the 4 participants who looked at both images.

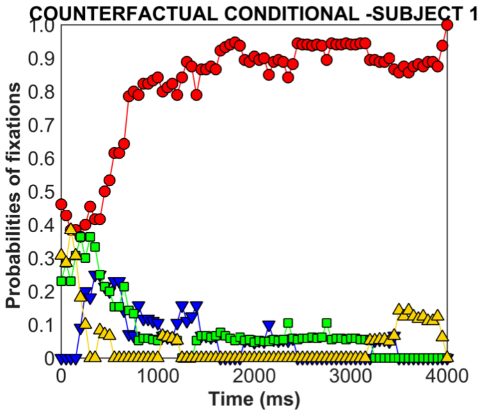

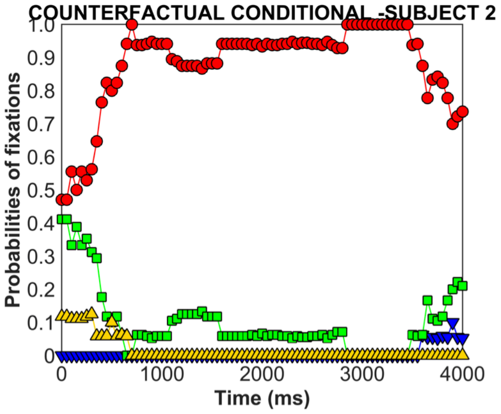

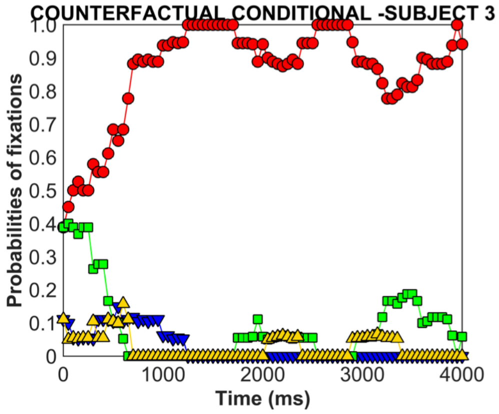

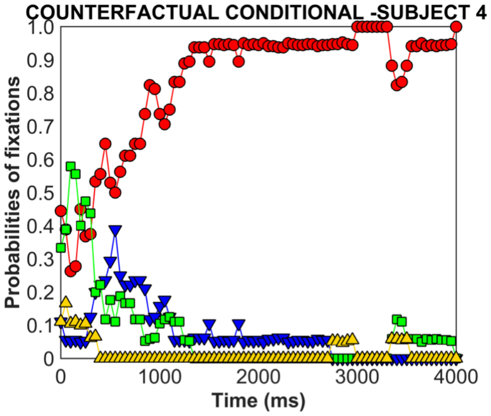

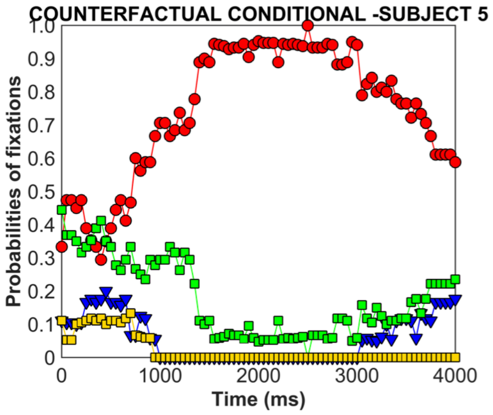

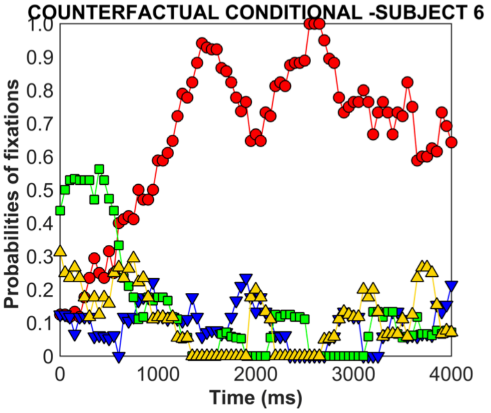

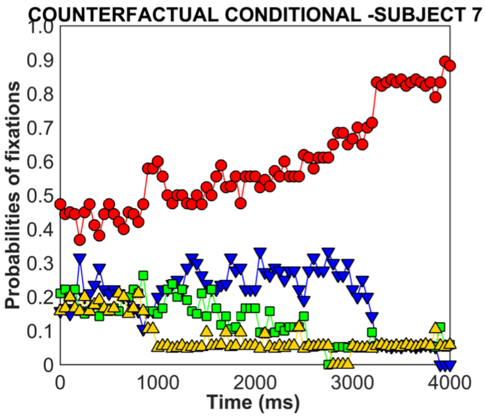
COUNTERFACTUAL CONDITIONAL -SUBJECT 7
Probabilities of fixations
Time (ms)

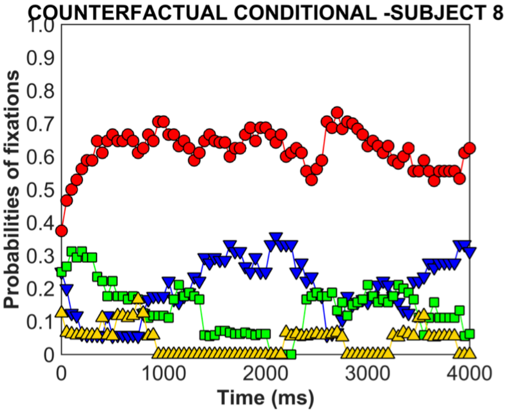
COUNTERFACTUAL CONDITIONAL -SUBJECT 8
Probabilities of fixations
Time (ms)

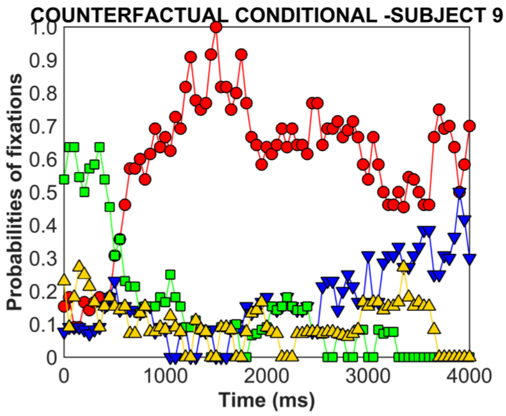
COUNTERFACTUAL CONDITIONAL -SUBJECT 9
Probabilities of fixations
Time (ms)

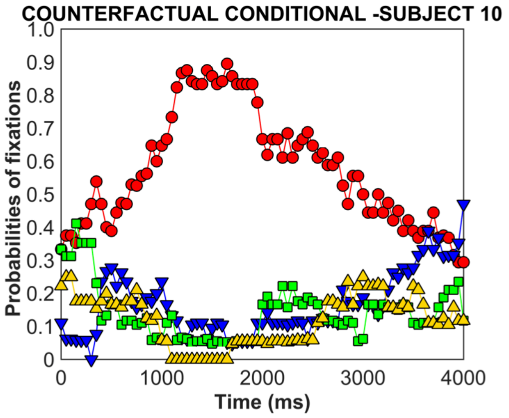
COUNTERFACTUAL CONDITIONAL -SUBJECT 10
Probabilities of fixations
Time (ms)

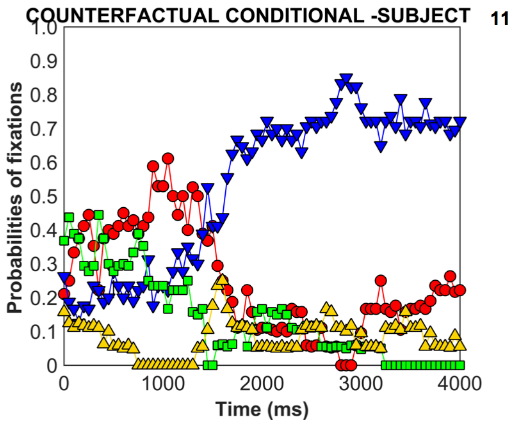
COUNTERFACTUAL CONDITIONAL -SUBJECT 11
Probabilities of fixations
Time (ms)

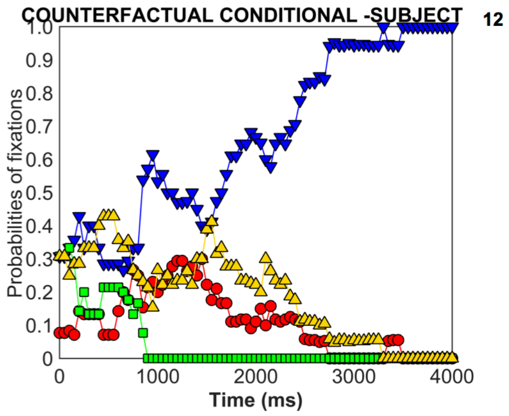
COUNTERFACTUAL CONDITIONAL -SUBJECT 12
Probabilities of fixations
Time (ms)

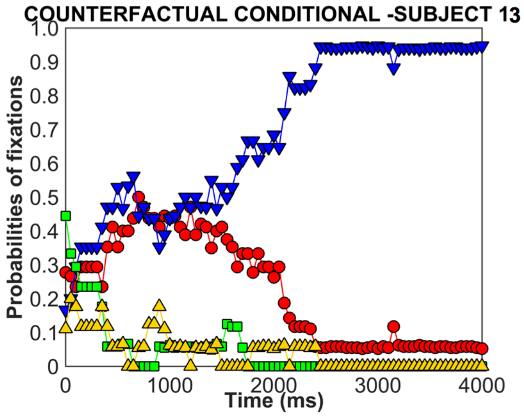
COUNTERFACTUAL CONDITIONAL -SUBJECT 13
Probabilities of fixations
Time (ms)

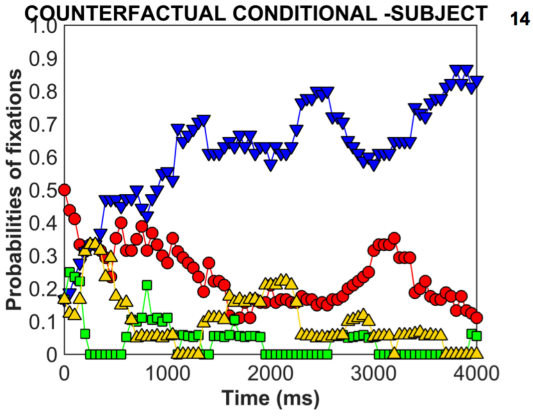
COUNTERFACTUAL CONDITIONAL -SUBJECT 14
Probabilities of fixations
Time (ms)

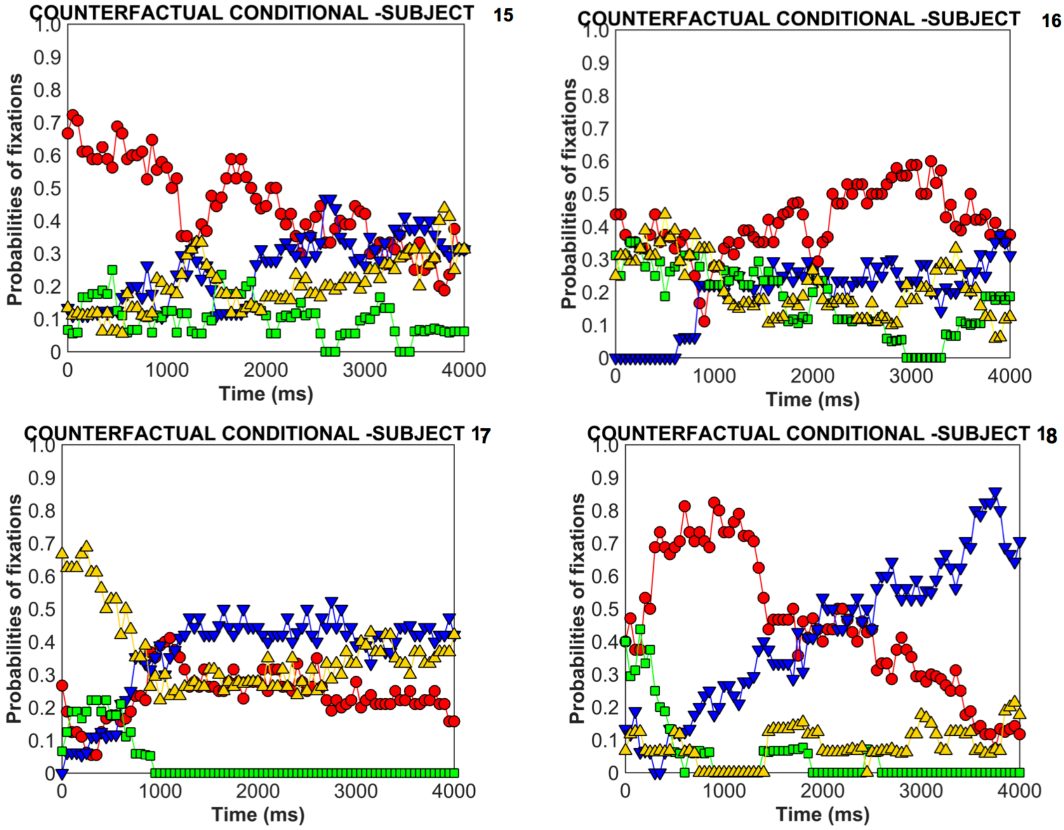
COUNTERFACTUAL CONDITIONAL -SUBJECT 15
COUNTERFACTUAL CONDITIONAL -SUBJECT 16
COUNTERFACTUAL CONDITIONAL -SUBJECT 17
COUNTERFACTUAL CONDITIONAL -SUBJECT 18
Probabilities of fixations
Time (ms)
0
0.1
0.2
0.3
0.4
0.5
0.6
0.7
0.8
0.9
1.0
1000
2000
3000
4000

Experiment 3

Probabilities of fixations for counterfactuals are presented for each individual participant in Experiment 3. The 9 participants who looked at the affirmative printed words only are presented first, then the 8 participants who looked at the negative printed words only, and then the 5 participants who looked at both.

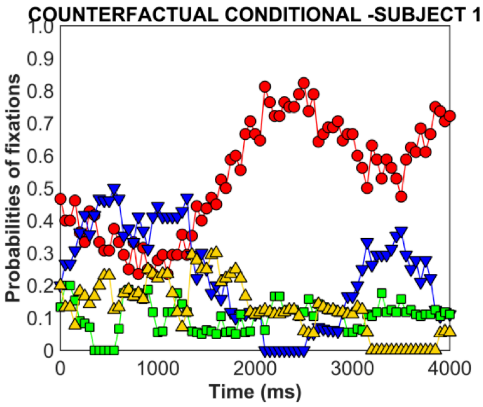

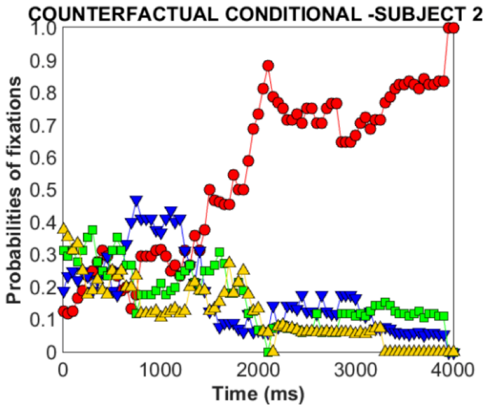

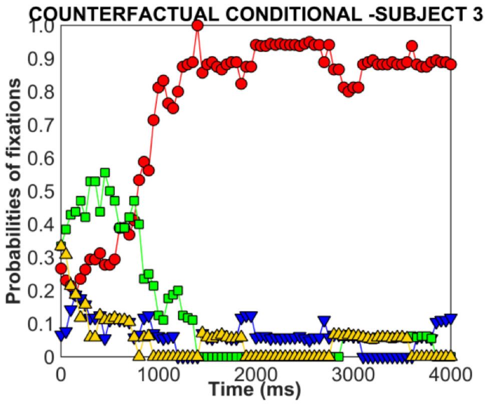

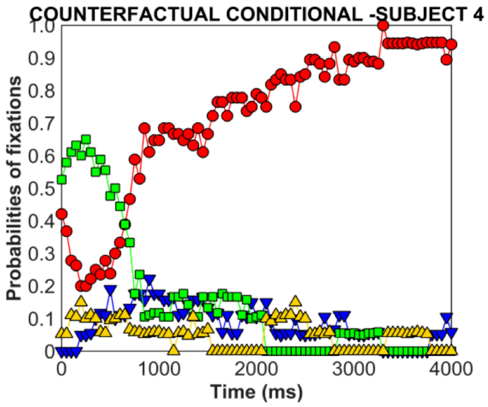

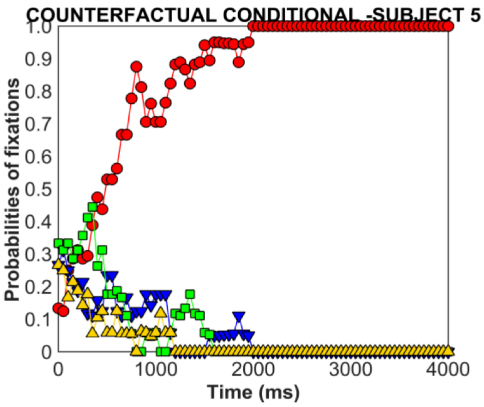

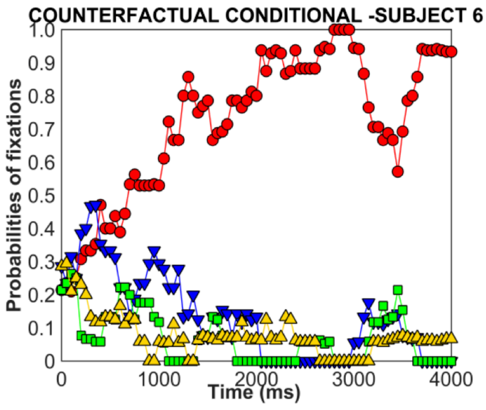

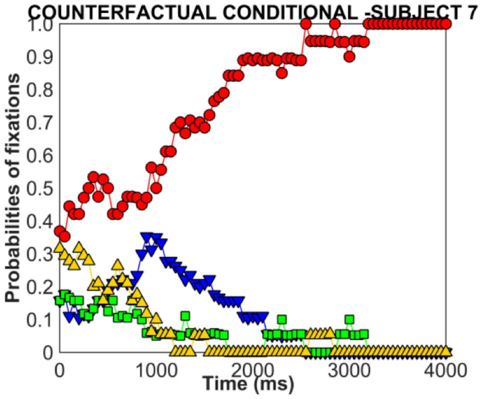
COUNTERFACTUAL CONDITIONAL -SUBJECT 7
Probabilities of fixations
Time (ms)

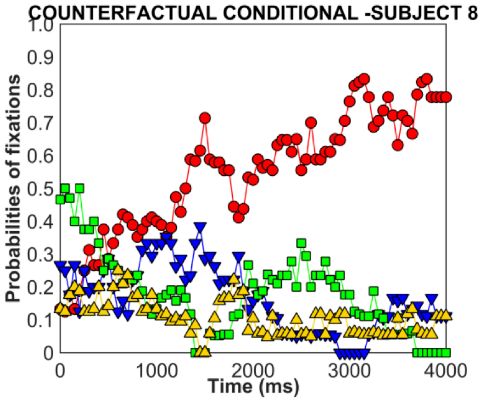
COUNTERFACTUAL CONDITIONAL -SUBJECT 8
Probabilities of fixations
Time (ms)

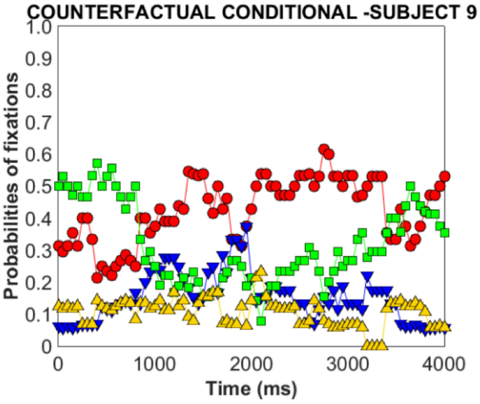
COUNTERFACTUAL CONDITIONAL -SUBJECT 9
Probabilities of fixations
Time (ms)

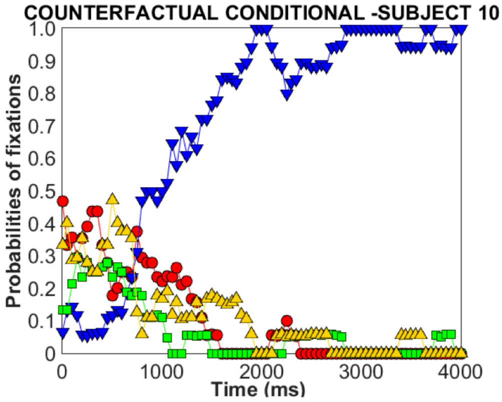
COUNTERFACTUAL CONDITIONAL -SUBJECT 10
Probabilities of fixations
Time (ms)

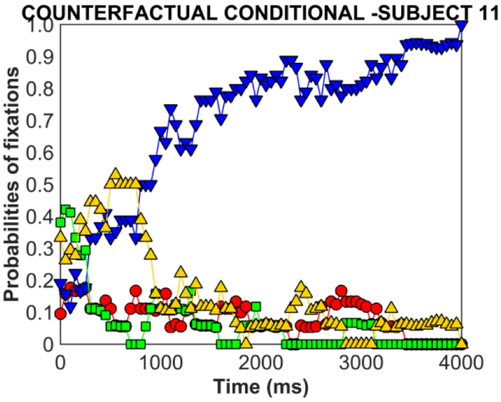
COUNTERFACTUAL CONDITIONAL -SUBJECT 11
Probabilities of fixations
Time (ms)

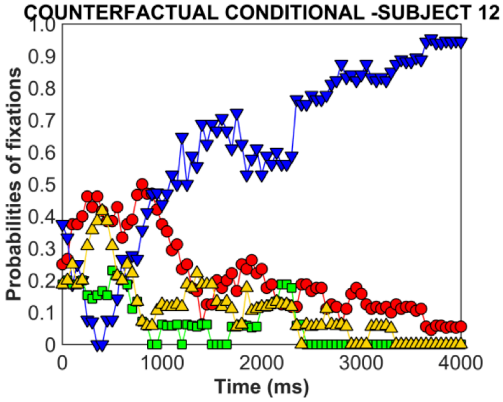
COUNTERFACTUAL CONDITIONAL -SUBJECT 12
Probabilities of fixations
Time (ms)

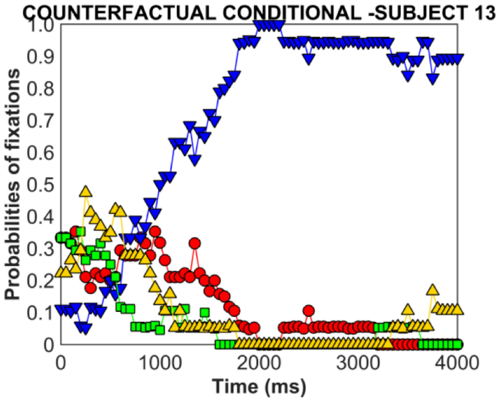
COUNTERFACTUAL CONDITIONAL -SUBJECT 13
Probabilities of fixations
Time (ms)

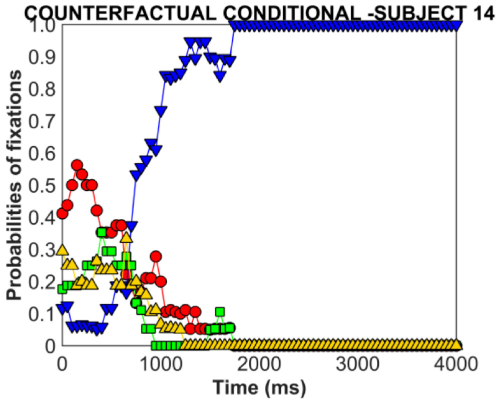
COUNTERFACTUAL CONDITIONAL -SUBJECT 14
Probabilities of fixations
Time (ms)

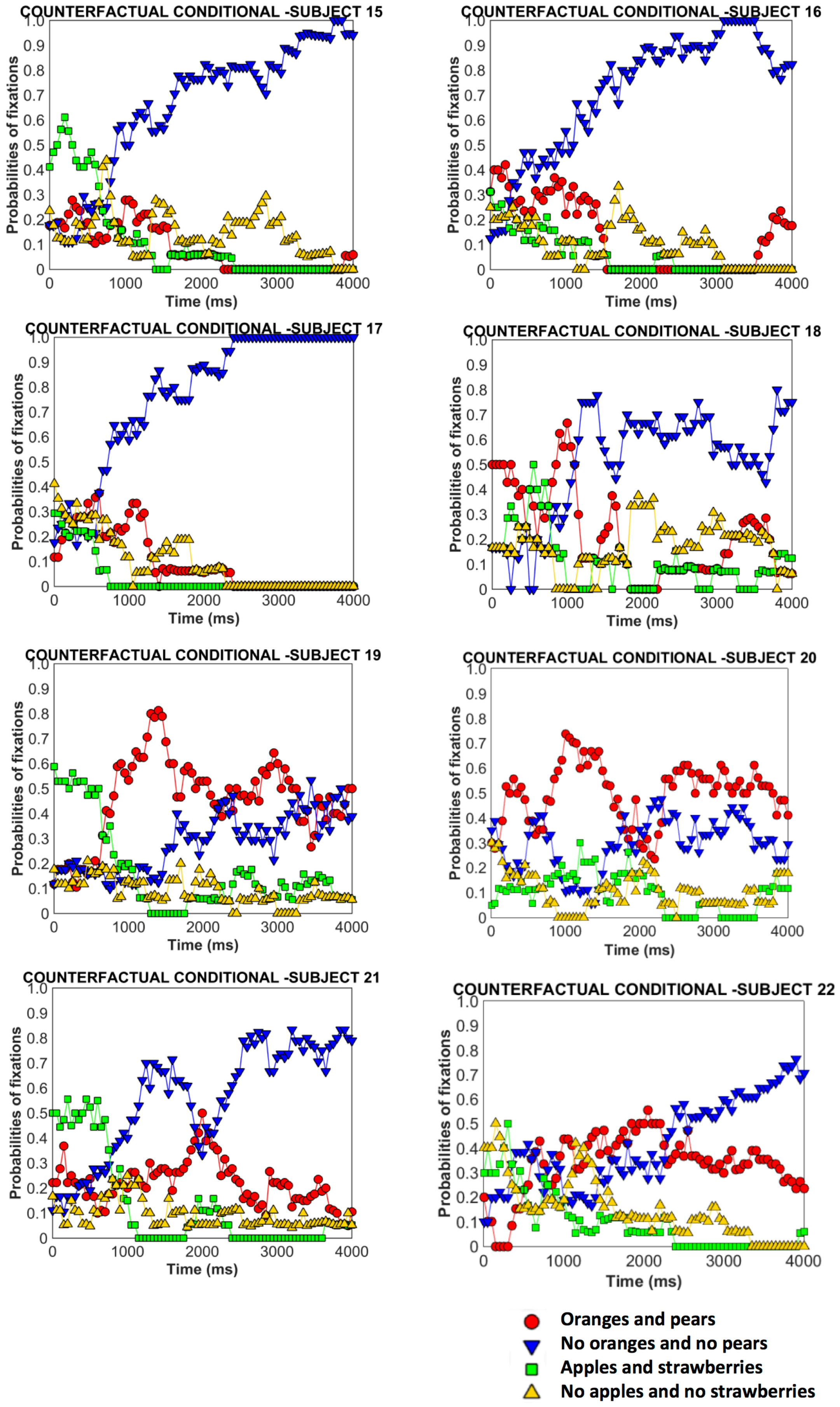
COUNTERFACTUAL CONDITIONAL -SUBJECT 15
COUNTERFACTUAL CONDITIONAL -SUBJECT 16
COUNTERFACTUAL CONDITIONAL -SUBJECT 17
COUNTERFACTUAL CONDITIONAL -SUBJECT 18
COUNTERFACTUAL CONDITIONAL -SUBJECT 19
COUNTERFACTUAL CONDITIONAL -SUBJECT 20
COUNTERFACTUAL CONDITIONAL -SUBJECT 21
COUNTERFACTUAL CONDITIONAL -SUBJECT 22
Probabilities of fixations
Time (ms)
Oranges and pears
No oranges and no pears
Apples and strawberries
No apples and no strawberries
